# Targeting the serotonergic system in the treatment of neurodegenerative diseases—emerging therapies and unmet challenges

**DOI:** 10.1016/j.pharmr.2025.100071

**Published:** 2025-06-02

**Authors:** Alina Brüge, Evgeni Ponimaskin, Josephine Labus

**Affiliations:** Cellular Neurophysiology, Hannover Medical School, Hannover, Germany

## Abstract

More than 65 million people worldwide experience neurodegenerative diseases, such as Alzheimer disease, frontotemporal dementia, Parkinson disease, and amyotrophic lateral sclerosis. As the risk of developing these diseases increases with age, increasing life expectancy will further accelerate their prevalence. Despite major advances in the understanding of the molecular mechanisms of neurodegeneration, no curative therapy is available to date.

Neurodegenerative diseases are known to be associated with alterations in serotonergic neurotransmission, which might critically contribute to the pathogenesis of these diseases. Therefore, targeting the serotonergic system appears to be a promising therapeutic approach.

In this review, we provide a comprehensive overview of pathological changes in serotonergic neurotransmission in different neurodegenerative diseases and discuss novel treatment strategies based on targeted modulation of the serotonergic system. We primarily focus on the therapeutic approaches modulating serotonin homeostasis, its biosynthesis, and the modulation of defined serotonin receptors.

**Significance Statement:**

A common feature of multiple neurodegenerative diseases is dysregulation of the serotonergic system at the cellular, molecular, and genetic levels that strongly contributes to specific pathological phenotypes. Targeting these alterations represents a suitable therapeutic strategy to combat disease-relevant pathomechanisms, slow down disease progression, and overcome pathological consequences.

## I Introduction

Serotonin (5-hydroxytryptamine, 5-HT) is an important monoamine that acts as a neurotransmitter and neuromodulator in the nervous system as well as a tissue hormone in the periphery. First isolated in 1937 from enterochromaffin cells as an inducer of smooth muscle and vessel contraction,^1^^,^^2^ it later became one of the most studied neurotransmitters in the central nervous system (CNS). 5-HT-mediated functions are diverse and include the regulation of anger, aggression, body temperature, appetite, sleep, mood, and pain.^3^, ^4^, ^5^, ^6^, ^7^ The serotonergic system is one of the earliest developed neuronal systems^8^ and serotonergic transmission is temporally and spatially tightly controlled, allowing for proper neuronal development and network formation.^9^ Not surprisingly, 5-HT imbalances are at the origin of many neurological and neuropsychiatric disorders. In the last decades, the serotonergic system has been intensively studied as a therapeutic target for depression, mood disorders, schizophrenia, and autism.^10^ In addition, the serotonergic system has recently gained attention as a potential target for the treatment of neurodegenerative diseases because dysfunction of the serotonergic system at the genetic, molecular, and cellular levels has been identified in several neurodegenerative diseases. Targeting these abnormalities has already been proposed as both a symptomatic and a disease-modifying treatment strategy for individual diseases.^11^, ^12^, ^13^

In this review, we provide a comprehensive overview of the role of the serotonergic system in major neurodegenerative diseases, with a particular focus on Alzheimer disease (AD), frontotemporal dementia (FTD), Parkinson's disease (PD), and amyotrophic lateral sclerosis (ALS). We begin by outlining disease-specific pathological phenotypes that are relevant to disease onset and progression, followed by a discussion of the serotonergic dysfunction characteristics of each disorder. In addition to summarizing current therapeutic approaches, we highlight specific strategies that target the serotonergic system and discuss their potential as converging therapeutic avenues across neurodegenerative diseases.

## II The serotonergic system

### A Biosynthesis and signaling

5-HT is a biogenic monoamine acting as a neurotransmitter and neuromodulator, as well as a peripheral hormone.^14^^,^^15^ 5-HT and its receptors are distributed in many organs and tissues of the body, including the brain, lungs, kidneys, platelets, and gastrointestinal tract. 5-HT regulates various physiological processes such as platelet aggregation, immune functions, vasoconstriction and vasodilation, appetite, circadian rhythm, nociception, body temperature, mood, as well as learning and memory.^3^^,^^4^^,^^6^^,^^7^^,^^16^, ^17^, ^18^, ^19^, ^20^, ^21^, ^22^, ^23^, ^24^, ^25^, ^26^, ^27^, ^28^, ^29^, ^30^, ^31^, ^32^, ^33^, ^34^, ^35^, ^36^

**Fig. 1:**
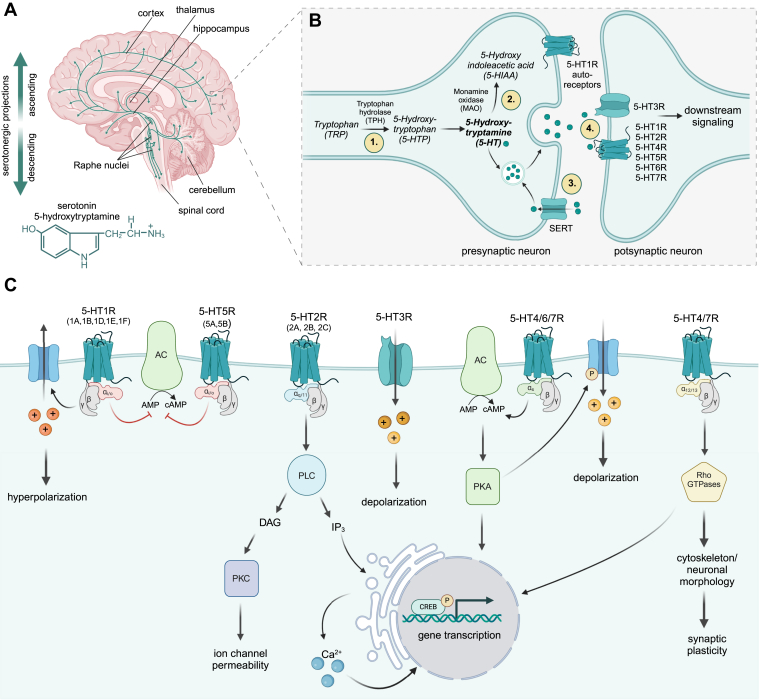
Serotonergic biosynthesis and signaling in the brain. (A) Serotonin is produced in small clusters of serotonergic neurons in the raphe nuclei. Ascending processes to the cortex, hippocampus, and thalamus, and descending processes to the cerebellum and spinal cord distribute 5-HT in the brain. (B) 5-HT is produced in a 2-step reaction from TRP, packed in vesicles, and released into the synaptic cleft upon depolarization of the presynaptic neuron. 5-HT can bind either on presynaptic autoreceptors to inhibit exocytosis of vesicles (negative feedback loop) or to postsynaptic receptors inducing downstream signaling in the postsynaptic neuron. Free 5-HT is re-taken up by SERT, followed by repacking into vesicles or degradation by MAO. Potential therapeutic targets to modulate the serotonergic system in the brain are (1) 5-HT biosynthesis, (2) 5-HT degradation, (3) 5-HT reuptake, and (4) modulation of 5-HTRs. (C) Simplified canonical signaling induced by binding of 5-HT to one of its 7 receptor families and corresponding subtypes. Apart from these pathways, 5-HTRs can signal also in a G protein-independent manner. AC, adenylate cyclase; CREB, cAMP response element-binding protein; DAG, diacylglycerol, 5-HIAA: 5 hydroxyindoleacetic acid; 5-HTP, 5-hydroxytryptophan; 5-HT, 5-hydroxytryptamines; 5-HTR, 5-hydroxytryptamine receptor or serotonin receptor; IP3, inositol 1,4,5-trisphosphate; PKA, protein kinase A; PKC, protein kinase C; PLC, phospholipase C; TRP, tryptophan.

Most of the body’s serotonin is produced in the gut by enterochromaffin cells and myenteric neurons.^37^, ^38^, ^39^ In the CNS, 5-HT is synthesized by small clusters of serotonergic neurons located in the raphe area of the pons and the midbrain.^40^ From there, serotonergic projections extend rostrally via ascending fibers to almost all brain regions, including the cortex, hippocampus, amygdala, thalamus, and striatum, or caudally via descending fibers to the spinal cord and the cerebellum (Fig. 1A).^10^^,^^41^ The amino acid L-tryptophan (TRP) is the precursor of 5-HT and is transformed to 5-hydroxytryptophan (5-HTP) by tryptophan hydroxylase (TPH). This rate-limiting step in serotonin biosynthesis is followed by the decarboxylation of 5-HTP to 5-HT by aromatic L-amino acid decarboxylase.^42^ Serotonergic axons branch abundantly and are characterized by numerous varicosities, in which 5-HT is stored in presynaptic vesicles, releasing into the synaptic cleft upon depolarization of the serotonergic neuron.^43^ Once released, 5-HT can bind to its presynaptic and postsynaptic receptors on neurons or act on neighboring cells such as astrocytes and microglia. Unbound 5-HT either undergoes reuptake by presynaptic 5-HT transporters or breakdown by monoamine oxidases (MAOs), resulting in the production of its metabolite 5-hydroxyindoleacetic acid (5-HIAA).^43^^,^^44^ A strict regulation of 5-HT synthesis, recycling, and catabolism is essential for proper brain function (Fig. 1B).

5-HT operates through the activation of a heterogeneous group of 5-HT receptors (5-HTRs) belonging to 7 different classes (5-HT1R to 5-HT7R), which differ in their molecular structure, cellular distribution, and signaling properties (Fig. 1C). Alternative splicing of some 5-HTR genes further enhances the complexity of the serotonergic receptor family, resulting in at least 14 different subtypes that have been identified in humans and 15 in mouse.^45^^,^^46^ Except for the 5-HT3R, all other 5-HTRs are members of the G protein-coupled receptor superfamily that induce intracellular signaling cascades by acting on a variety of heterotrimeric G proteins.^47^

In contrast to other serotonergic receptors, 5-HT1Rs are expressed on both presynaptic and postsynaptic neurons. Presynaptic 5-HT1AR and 5-HT1BR act as autoreceptors, which, when activated, provide negative feedback signals to control the release of 5-HT from the serotonergic neurons.^48^ Postsynaptic 5-HT1Rs as well as the 5-HT5Rs activate the G_i/o_ protein, which blocks the adenylate cyclase (AC), converting adenosine monophosphate (AMP) to cyclic AMP (cAMP). Furthermore, the 5-HT1R-mediated activation of the G_*β*/*γ*_ subunit stimulates the opening of inwardly rectifying potassium channels and calcium-dependent potassium channels, and inhibits calcium channels, resulting in hyperpolarized postsynaptic potentials.^49^, ^50^, ^51^, ^52^, ^53^ Stimulation of 5-HT2R subtypes activates the G_q/11_ signaling pathway, in which phospholipase C catalyzes the hydrolysis from phosphatidylinositol 4,5-bisphosphate to inositol 1,4,5-trisphosphate and diacylglycerol.^54^ Inositol 1,4,5-trisphosphate stimulates the release of calcium from the endoplasmic reticulum, which contributes to the activation of the transcription factor cAMP response element-binding protein (CREB) and the regulation of gene expression.^55^^,^^56^ Diacylglycerol activates the protein kinase C, which inhibits sodium channels and modulates neuronal excitability.^57^ As mentioned above, signaling of the 5-HT3R does not involve G proteins and second messengers. As a cation-selective ligand-gated ion channel, 5-HT3R mediates the influx of sodium, potassium, and calcium ions, resulting in depolarization of the postsynaptic neuron.^58^^,^^59^ The receptor families 5-HT4R, 5-HT6R, and 5-HT7R are coupled to the heterotrimeric G_s_ protein, which stimulates the AC, resulting in increased cAMP production. Subsequently, the protein kinase A (PKA) is activated, which in turn modulates gene transcription by phosphorylation of CREB.^60^ Moreover, PKA can regulate the activity of several ion channels by phosphorylation, eg, calcium channels and G protein-coupled inwardly rectifying potassium channels, thereby contributing to the excitatory effects of these receptors.^61^, ^62^, ^63^, ^64^, ^65^, ^66^

In addition to these canonical G protein pathways, 5-HT4Rs and 5-HT7Rs have been shown to couple to G_12/13_ proteins involved in the regulation of small GTPases of the Rho family.^67^, ^68^, ^69^, ^70^ Several 5-HTRs also act in a G protein-independent manner via the extracellular signal-regulated kinase (ERK), mammalian target of rapamycin, and cyclin-dependent kinase 5 (Cdk5), thereby modulating gene transcription and synaptic plasticity in neurons.^9^^,^^71^^,^^72^

### B The role of the serotonergic system in neurodegenerative diseases

A tight spatial and developmental regulation of serotonergic signaling is critical for multiple brain functions. Not surprisingly, serotonergic imbalances have been implicated in a wide range of neuropsychiatric and neurological disorders, including schizophrenia, anxiety, depression, autism, migraine, and addiction.^10^

In recent years, the serotonergic system has also gained attention in the research of the pathogenesis of neurodegenerative diseases, a heterogeneous group of disorders characterized by progressive neuronal loss and dysfunction. Among the most prominent members are AD, FTD, PD, and ALS, which affect more than 65 million people worldwide. They differ markedly in disease progression and the spectrum of clinical symptoms that range from cognitive impairment and personality changes to severe motor dysfunction.^73^

At the molecular level, AD, FTD, PD, and ALS are classified as proteinopathies, in which misfolding, aggregation, and deposition of various proteins are common. The predominant misfolded proteins found in AD are amyloid-*β* (A*β*) and tau, in FTD tau and TAR DNA-binding protein 43 (TDP-43), in PD *α*-synuclein (*α*-syn), and in ALS TDP-43.^74^, ^75^, ^76^, ^77^, ^78^ Although in their native forms, these proteins differ considerably in size, subcellular localization, and function, they can all aggregate into large fibrils called amyloids. Recent evidence suggests that smaller intermediates, such as misfolded oligomers, rather than the large amyloids as originally thought, are the predominant neurotoxic species.^79^ Their ability to self-propagate and spread across anatomically linked brain regions like prion proteins contributes to the progressive nature of neurodegenerative diseases.^80^

Protein misfolding and aggregation have long been considered the primary driving force in the process of neurodegeneration, but over the past decades, there has been substantial progress in our understanding of the underlying molecular mechanisms. Nowadays, it is widely accepted that AD, FTD, PD, and ALS are multifactorial diseases. These are driven by a complex interplay between various genetic and molecular pathways, causing pathological protein aggregation, DNA and RNA defects, synaptic and mitochondrial dysfunction, cytoskeletal abnormalities, defective proteostasis, and neuroinflammation.^81^ In addition to genetic predisposition and the susceptibility of certain genes, environmental factors and lifestyle choices such as exposure to chemicals and pesticides, strenuous exercise, diet, and smoking are crucial in the development of neurodegenerative diseases.^81^, ^82^, ^83^, ^84^

**Fig. 2:**
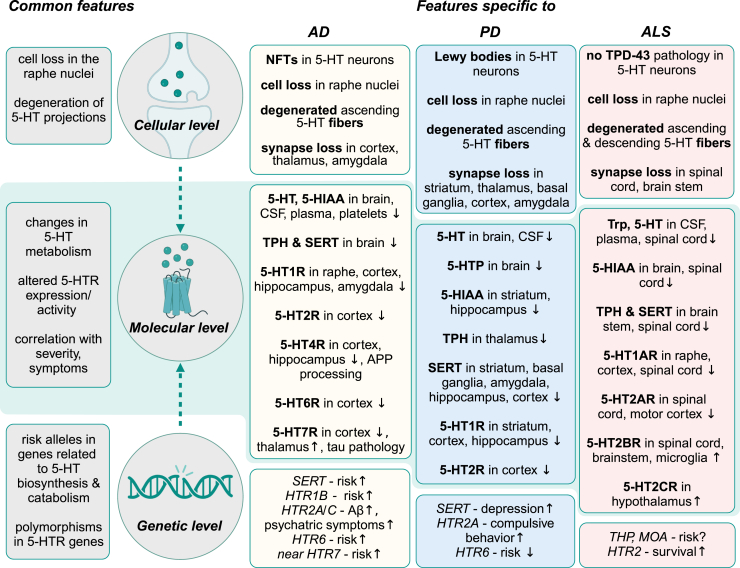
Common and disease-specific serotonergic abnormalities observed in AD, PD, and ALS. Serotonergic imbalances are common in neurodegenerative diseases at the cellular, molecular, and genetic levels and manifest in specific abnormalities characteristic of AD, PD, and ALS.

**Fig. 3:**
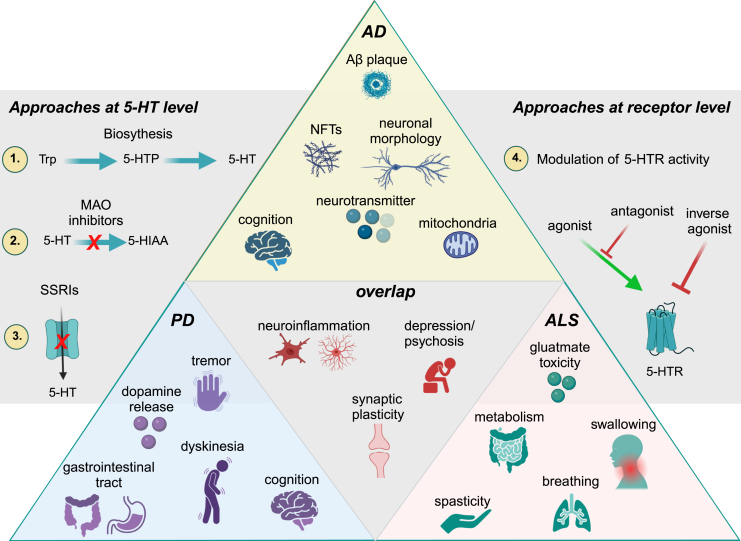
Overview of proposed interventions targeting the serotonergic system in neurodegenerative diseases. Proposed approaches to modulate the serotonergic neurotransmission at the ligand and receptor level to target common disease-relevant mechanisms and associated symptoms in several neurodegenerative diseases (gray) or individual disorders (AD/FTD – yellow, PD – blue, ALS – red). Numbers 1. - 4. introduce possible therapeutic approaches to modulate the serotonergic system as follows: 1. promoting 5-HT biosynthesis, 2. preventing 5-HT degradation by MAOIs, 3. blocking 5-HT reuptake by SSRIs, and 4. modifying receptor activity by 5-HTR ligands.

Dysfunction of 5-HT-related processes, including 5-HT synthesis, release, reuptake, receptor activity, and gene expression, has been identified in several neurodegenerative diseases, including AD and FTD, PD, and ALS (Fig. 2). Consequently, the 5-HT system has emerged as a novel potential target in preclinical and clinical drug development. Several approaches have been proposed to specifically modulate the serotonergic system, suggesting either disease-modifying effects or amelioration of associated symptoms. These strategies include (1) enhancing 5-HT biosynthesis, eg, by supplementation of its chemical precursors; (2) reducing 5-HT reuptake via selective serotonin reuptake inhibitors (SSRIs); (3) decreasing 5-HT degradation using monoamine oxidase inhibitors (MAOIs); and (4) therapeutically modulating the activity of defined 5-HTRs to normal levels (Fig. 1B; Fig. 3).

## III Alzheimer disease and other dementias

### A Disease-specific pathological phenotypes

Dementia is a heterogeneous group of diseases affecting more than 55 million people worldwide (Table 1).^85^, ^86^, ^87^, ^88^, ^89^, ^90^, ^91^, ^92^, ^93^, ^94^, ^95^, ^96^, ^97^, ^98^, ^99^, ^100^, ^101^, ^102^, ^103^, ^104^, ^105^, ^106^, ^107^, ^108^, ^109^ The symptoms of these diseases can vary greatly among individual patients and include progressive deficits in cognition, executive functions, learning and memory, and language, as well as behavioral and psychological changes, including depression, psychosis, anxiety, and aggressiveness.^92^^,^^93^ AD is the most common cause of dementia, accounting for 60%–70% of all cases, with women being disproportionately highly affected.^86^^,^^110^ Rarer forms of dementia include vascular dementia (5%–30%), dementia with Lewy bodies (10%–15%), and FTD (10%–15%).^111^, ^112^, ^113^, ^114^ FTD is characterized by the progressive degeneration of the frontal and temporal lobes, with clinical onset typically occurring before the age of 65 years. In addition to age as the primary risk factor for developing dementia, genetics significantly contribute to disease development (Table 1).

**Table 1:** Overview of pathological phenotypes in AD, PD, and ALS

	AD and FTD^a^	PD^b^	ALS^c^	References
Epidemiology	AD: > 40 million women > men FTD: > 1 million men > women	> 10 million men > women	< 200,000 men > women	^a^^85^, ^86^, ^87^ ^b^^88^, ^89^, ^90^ ^c^^91^
Symptoms	cognitive decline, executive dysfunction, learning and memory impairment, behavioral changes, language impairment, depression	bradykinesia, rigidity, tremor depression, cognitive decline, psychosis, pain, sleep disturbances, gastrointestinal dysfunction	muscle atrophy, spasticity, paralysis, dysarthria, dysphagia, weight loss, fatigue, emotional lability, cognitive dysfunction	^a^^92^^,^^93^ ^b^^94^^,^^95^ ^c^^96^
Affected areas	neocortex, limbic system, cerebellum, brainstem	nigrostriatal system (substantia nigra and dorsal striatum)	motor neurons in the motor cortex, brainstem, spinal cord	^a^^80^^,^^97^ ^b^^95^ ^c^^98^
Protein aggregates	AD: A*β*, tau FTD: tau, TDP-43, FUS	a-Synuclein	TDP-43, FUS, SOD1, C9ORF-related polypeptide	^a^^74^^,^^76^^,^^77^ ^b^^78^ ^c^^77^^,^^99^, ^100^, ^101^, ^102^
Deterministic genes	AD:*APP*, *PSEN-1*, *PSEN-2* FTD:*MAPT*, *TARDBP*, *C9ORF*, *GRN*	*GBA, SNCA, LRRK2, PRKN*, *PINK1, DJ-1*	*TARDBP*, *FUS*, *SOD1*, *C9ORF*	^a^^103^^,^^104^ ^b^^105^ ^c^^82^^,^^106^
Pathomechanisms	cholinergic and other neurotransmitter deficits, protein aggregation, mitochondrial and lysosomal dysfunctions, vesicle trafficking impairment, synaptic deficits, oxidative stress, neuroinflammation	dopaminergic deficits, protein aggregation, mitochondrial and lysosomal dysfunctions, vesicle trafficking impairment, synaptic deficits, oxidative stress, neuroinflammation	glutamatergic excitotoxicity, protein aggregation, mitochondrial and autophagic dysfunctions, DNA/RNA defects, stress granule formation, oxidative stress, neuroinflammation	^a^^107^^,^^108^ ^b^^83^ ^c^^96^^,^^98^^,^^109^

Although AD and FTD share similar clinical phenotypes, especially in the early stages of the disease, the underlying pathophysiological mechanisms differ significantly. Extracellular plaques composed of A*β* peptides are the characteristic hallmark of AD pathogenesis.^76^ Pathological A*β* species are generated by the amyloidogenic cleavage of the amyloid-*β* precursor protein (APP) protein by the *β*-secretase *β*-site amyloid precursor protein cleaving enzyme 1 and the *γ*-secretase.^115^ Besides their ability to self-aggregate, A*β* oligomers have been shown to bind to several neurotransmitter receptors, leading to excessive calcium influx, oxidative stress, inflammation, mitochondrial and synaptic dysfunction, and ultimately neuronal death.^116^, ^117^, ^118^, ^119^, ^120^, ^121^ AD is also characterized by the presence of intracellular neurofibrillary tangles (NFTs) composed of the microtubule-binding protein tau.^74^ Tau hyperphosphorylation, A*β*-mediated toxicity, and oxidative stress trigger the detachment of tau from microtubules, promoting the formation of tau aggregates.^122^, ^123^, ^124^, ^125^, ^126^, ^127^, ^128^ Although the pathogenic role of tau aggregates in neurotoxicity is not fully understood yet, tau aggregation is most likely associated with a loss of tau’s physiological function in stabilizing microtubules, resulting in cytoskeleton abnormalities, impaired axonal transport, synaptic dysfunction, and subsequent neurodegeneration.^129^, ^130^, ^131^, ^132^, ^133^, ^134^

In patients with FTD, aggregates of tau and the DNA/RNA-binding proteins TDP 43 and fused in sarcoma (FUS) are present.^77^ TDP-43 is primarily localized in the nucleus and controls neuronal functions by regulating RNA metabolism. Mutations in the *TARDBP* gene and posttranslational modifications, such as phosphorylation and ubiquitination, facilitate the mislocalization of TDP-43 to the cytosol and its aggregation into insoluble inclusions.^135^, ^136^, ^137^, ^138^, ^139^, ^140^, ^141^ In addition to the cytotoxic properties of these aggregates, the loss of functional TDP-43 in the nucleus promotes neuronal dysfunction and degeneration.^142^, ^143^, ^144^, ^145^ Similar pathogenic mechanisms have been proposed for FUS, which is critically involved in cell proliferation, DNA double-strand break repair, RNA splicing, and transport.^146^, ^147^, ^148^, ^149^ Of note, the deposition of *α*-syn typical of PD has also been observed in both AD and FTD.^150^, ^151^, ^152^

Besides the presence of protein aggregates, neuroinflammation is another prominent pathological hallmark of AD and FTD. Chronic activation of astrocytes and microglia during disease progression initiates maladaptive responses, such as changes in morphology and secretion of proinflammatory cytokines, resulting in the production of reactive oxygen species (ROS), neuronal death, and increased plaque burden.^153^

**Fig. 4:**
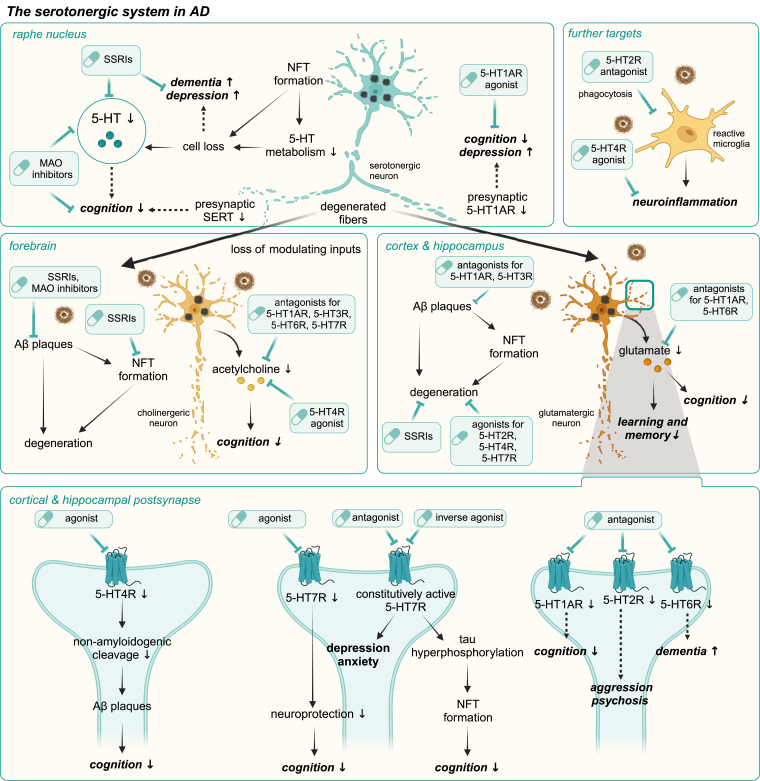
Serotonergic deficiencies in AD and its therapeutic targeting. Overview of serotonergic changes in the brain during the progression of AD. Degeneration of the serotonergic system occurs early in the course of the disease. Pronounced NFT formation drives the degeneration of serotonergic neurons in the raphe nuclei (upper left panel), while A*β* plaques were found only occasionally in this region. As a result, the dramatic loss of serotonin-producing cells and impaired 5-HT metabolism lead to reduced 5-HT levels, which correlate with the severity of dementia, cognitive decline, and depressive symptoms. Furthermore, degeneration of ascending serotonergic fibers and reduced levels of SERT and presynaptic 5-HT1AR are found in patients with AD. These changes may contribute to the A*β* and NFT-driven degeneration of cholinergic neurons in the forebrain (middle left panel) and glutamatergic neurons in the cortex and hippocampus (middle right panel). The loss of modulating serotonergic input, both excitatory and inhibitory, causes an imbalance in 5-HTR expression in these brain regions, which are critically involved in cognition, learning, and memory, as well as the development of dementia-associated symptoms such as depression, psychosis, and behavioral changes. Furthermore, the 5-HT4R and 5-HT7R play a pivotal role in A*β* processing and tau hyperphosphorylation (lower panel), indicating that alterations in their expression and/or activity directly impact the progression of AD pathology. In addition to neuronal degeneration, reactive microglia drive neuroinflammation in the affected brain regions, further exacerbating disease progression (upper right panel). The potential direct consequences of serotonergic alterations are indicated by arrows with solid lines, and correlations by arrows with dashed lines. Proposed targeted interventions for serotonergic imbalances are highlighted by green boxes with a pill symbol.

Disturbances in various neurotransmitter systems have been implicated in the pathogenesis of AD and FTD.^154^, ^155^, ^156^, ^157^, ^158^, ^159^, ^160^, ^161^, ^162^, ^163^, ^164^, ^165^, ^166^, ^167^, ^168^, ^169^, ^170^, ^171^, ^172^, ^173^, ^174^, ^175^ Among these, the cholinergic system is particularly impaired in AD,^176^, ^177^, ^178^, ^179^^,^ and may contribute to FTD pathology.^180^^,^^181^ In addition to cholinergic dysfunction, alterations in other neurotransmitter systems, including the adrenergic, glutamatergic, and dopaminergic pathways, have been observed. Importantly, growing evidence also points to a critical involvement of the serotonergic system in the development and progression of dementia (Fig. 4).

### B Serotonergic imbalances

Since the early 1980s, several independent research groups have observed a dramatic loss of serotonergic neurons, ranging between 20% and 40%, in the dorsal raphe nuclei in postmortem tissue from patients with AD.^182^, ^183^, ^184^, ^185^, ^186^ More recent studies have confirmed these findings, indicating a disease-specific predominance of non-5-HT neurons in the raphe nuclei in AD.^187^, ^188^, ^189^ While A*β* plaques were only occasionally found in this region, NFT formation was pronounced and correlated with an increased loss of serotonergic neurons in the dorsal raphe nuclei in individual patients.^183^^,^^185^^,^^187^ It was further suggested that increased serotonergic cell loss is associated with the severity of dementia and depressive symptoms.^183^^,^^186^ Reduced 5-HT1AR densities in the raphe nuclei of patients with AD quantified using positron emission tomography (PET) further support the loss of central serotonergic neurons.^190^ Degeneration of serotonergic neurons was not only obvious in the raphe nuclei but also in the cortex. Autoradiography with [^3^H]-imipramine, a selective serotonin transporter (SERT) inhibitor, revealed reduced binding in the putamen and frontal, temporal, and cingulate cortex, indicating the loss of presynaptic 5-HT terminals.^191^, ^192^, ^193^ Decreased SERT binding was also observed in different brain areas, including the cortical, limbic, sensory, and motor regions in patients with mild cognitive impairment using magnetic resonance (MR) imaging and PET, which correlated with impaired auditory-verbal and visual-spatial memory in these patients.^194^^,^^195^ Studies in animal models of AD show partial consistency with human data. Serotonergic degeneration has been observed in the dorsal and median raphe nuclei, as well as in cortical and hippocampal regions across various transgenic animal models.^196^^,^^197^ However, other findings indicate no significant loss of serotonergic neurons in the raphe nuclei, but rather an increase in hippocampal SERT-positive fibers.^198^ These discrepancies highlight the complexity of serotonergic alterations in AD models and indicate the need for further comparative studies.

Consistent with the serotonergic denervation in AD, reduced concentrations of 5-HT and its metabolite 5-HIAA have been observed in several brain regions, eg, the amygdala, putamen, thalamus, globus pallidus, inferior temporal gyrus, and frontal and temporal cortex of patients with AD and animal models of AD, indicating not only serotonergic cell loss but also diminished metabolic activity in serotonergic neurons.^192^^,^^199^, ^200^, ^201^, ^202^, ^203^, ^204^ Similar observations have been made in the cerebrospinal fluid (CSF) and blood platelets of patients with AD.^172^^,^^173^^,^^205^, ^206^, ^207^ In the frontal cortex and CSF, reduced 5-HT levels also correlated with the severity of cognitive decline.^173^^,^^208^ Furthermore, decreased activity of TPH, the rate-limiting enzyme in 5-HT biosynthesis, has been shown in various brain regions, such as the globus pallidus lateral segment, locus coeruleus, and substantia nigra of patients with AD.^209^ It has been suggested that impaired 5-HT metabolism may be a consequence of pronounced tau pathology, resulting in cellular degeneration.

Serotonergic neurotransmission in AD is also impaired at the postsynaptic level. An early study from Bowen et al^191^ reported reduced [^3^H]-serotonin binding in the frontal and temporal cortex in postmortem AD samples. In line with this, numerous studies demonstrate the loss of postsynaptic 5-HTRs that might contribute to AD pathogenesis and symptom severity. Reduced 5-HT1AR densities in the hippocampus, entorhinal cortex, and amygdala of AD patients have been observed using PET imaging.^190^^,^^210^, ^211^, ^212^, ^213^ Similar observations have been reported for 5-HT1BRs and 5-HT1DRs in the frontal and temporal cortex of postmortem AD samples.^214^ While some studies found a correlation between reduced 5-HT1AR density and deterioration of clinical symptoms as well as aggressive behavior, other studies correlated increased 5-HT1AR as well as 5-HT1B/D densities with greater cognitive decline.^190^^,^^208^^,^^210^^,^^214^, ^215^, ^216^ In contrast, Borg et al^217^ reported no correlation between 5-HT1R expression and cognitive function.^217^ Additionally, a polymorphism in the *HTR1B* gene has been associated with a higher *APOE4* genotype frequency and deteriorated performance in neuropsychological tests.^218^ Furthermore, decreased levels of 5-HT2AR have been observed in the neocortex of AD patients using single-photon emission computed tomography (SPECT).^219^ These findings have been supported by other studies demonstrating reduced 5-HT2AR densities in the neocortex, frontal, and temporal cortex of postmortem AD samples, which correlate with cognitive impairment.^194^^,^^220^^,^^221^ In addition, polymorphisms in the *HTR2A* and *HTR2C* genes correlate with the number of *APOE4* allele carriers and A*β* levels in the CSF.^218^ They are also associated with neuropsychiatric symptoms in AD, including depression, hallucinations, delusions, psychosis, aggression, motor deficits, and apathy.^222^, ^223^, ^224^, ^225^, ^226^, ^227^ Reduced 5-HT4R levels have been detected in the hippocampus and frontal cortex of postmortem AD samples.^228^ Constitutive 5-HT4R activity is involved in nonamyloidogenic APP cleavage by direct interaction with the *α*-secretase ADAM10, resulting in the production of sAPP*α*, which has neuroprotective effects.^229^, ^230^, ^231^, ^232^ Loss of 5-HT4R may therefore directly contribute to the pathogenesis of AD. Decreased 5-HT6R levels in the frontal and temporal cortex of patients with AD have been associated with cognitive, behavioral, and psychological symptoms of dementia.^214^^,^^221^ In addition, an *HTR6* polymorphism has been identified as a risk factor for developing AD and is associated with increased *APOE4* allele frequency as well as cognitive deficits.^218^^,^^233^ Expression of the 5-HT7R is also affected in AD. While mRNA levels were significantly increased in the thalamus, reduced levels have been observed in the prefrontal cortex.^234^ Furthermore, a single-nucleotide polymorphism (SNP) located near the *HTR7* gene has been identified in a Dutch population of patients with late-onset AD, and constitutive activity of this receptor has been implicated in the hyperphosphorylation of tau.^72^^,^^235^ Altered expression of postsynaptic 5-HTRs has been observed in various transgenic AD animal models, including APP/PS1 mice, 3×Tg-AD mice, Tg2576 mice, and humanized Tau mice, highlighting that AD is associated with alterations in serotonergic neurotransmission. However, the extent and nature of these alterations vary across different models, underscoring the need for further comparative studies to clarify model-specific differences and their relevance to human pathology.^236^, ^237^, ^238^, ^239^, ^240^, ^241^, ^242^

To date, it remains unclear whether serotonergic denervation in AD is just another aspect of widespread neurodegeneration of neurotransmitter systems or might be a causative factor contributing to disease onset and progression. Support for the latter hypothesis comes from the fact that NFT formation occurs in the raphe nucleus and has also been identified in nondemented controls, suggesting that serotonergic neurons are an early primary site for NFT formation.^187^ In line with this, serotonergic denervation is already evident at early disease stages and has been shown to precede the loss of cholinergic neurons.^182^^,^^191^ Furthermore, specific 5-HTRs, in particular the 5-HT4R and 5-HT7R, are involved in A*β* processing and tau hyperphosphorylation, suggesting that changes in their expression and/or activity directly contribute to pathology. Polymorphisms in the promoter region of the *SERT* gene and several *HTR* genes are associated with higher disease risk, further suggesting a causative role of the serotonergic system in AD^218^^,^^235^^,^^243^

Imbalances in the serotonergic system have also been implicated in FTD. However, in this case, studies are less consistent than for AD. Although Yang et al^244^, ^245^, ^246^, ^247^ have shown a 40% reduction of serotonergic neurons in the dorsal raphe nuclei as well as degeneration of ascending fibers to the forebrain using the graph method, other studies did not confirm presynaptic deficiencies. In line with normal [^3^H]-imipramine binding, levels of 5-HT and its metabolite 5-HIAA were unchanged in the cortex and CSF of patients with FTD.^172^^,^^207^^,^^244^^,^^245^^,^^248^ Reduced [^3^H]-serotonin binding has been observed in the cortex and hypothalamus, suggesting that serotonergic deficits in FTD are more likely to manifest at the postsynaptic level.^246^ In addition, decreased levels of 5-HT1AR and 5-HT2AR were found mainly in the frontal and temporal cortex of postmortem FTD samples.^244^^,^^249^ Consistent with this, PET imaging revealed a significant reduction of the 5-HT1AR and 5-HT2AR in cortical regions.^250^^,^^251^ It has been suggested that the preservation of serotonergic afferents observed in FTD might contribute to increased levels of extraneuronal 5-HT. Because 5-HT has inhibitory effects on pyramidal neurons, such elevated levels might lead to the underactivity of surviving glutamatergic neurons in the cortex. As a compensatory mechanism, postsynaptic 5-HT1R and 5-HT2R may be downregulated during FTD progression.^244^

Taken together, multiple studies provide evidence for the dysfunction of the serotonergic system in AD and FTD. While impaired serotonergic neurotransmission is evident at both the presynaptic and postsynaptic levels in patients with AD, postsynaptic 5-HTRs seem to be mainly affected by FTD. This finding underlines the fact that the mechanisms driving serotonergic imbalances in AD and FTD may differ, which in turn manifest in distinct symptoms.

### C Current treatment strategies

Current treatment strategies mainly target cholinergic and glutamatergic deficits. To date, the US Food and Drug Administration has approved 8 drugs for the treatment of AD (Table 2),^252^, ^253^, ^254^, ^255^, ^256^, ^257^, ^258^, ^259^, ^260^, ^261^, ^262^, ^263^, ^264^, ^265^, ^266^, ^267^, ^268^, ^269^, ^270^, ^271^, ^272^, ^273^, ^274^, ^275^, ^276^, ^277^, ^278^, ^279^, ^280^, ^281^, ^282^, ^283^, ^284^, ^285^, ^286^, ^287^, ^288^, ^289^, ^290^, ^291^, ^292^, ^293^, ^294^, ^295^, ^296^ but none for FTD. However, off-label use of AD medications is common in FTD therapy. One group of these drugs includes cholinesterase (ChE) inhibitors, targeting either acetylcholinesterase (AChE) and/or butyrylcholinesterase (BChE), thereby helping to restore the cholinergic neurotransmission.^252^, ^253^, ^254^ In addition to the cholinergic effects, ChEIs have neuroprotective effects by increasing A*β* phagocytosis and reducing glutamate neurotoxicity, A*β*-mediated toxicity, oxidative stress, neuroinflammation, and memory deficits in vitro as well as in vivo.^255^, ^256^, ^257^, ^258^, ^259^, ^260^, ^261^, ^262^, ^263^, ^264^, ^265^, ^266^ However, the disease-modifying effects of these drugs on cognition and behavior in patients with AD are still controversial.^267^, ^268^, ^269^

**Table 2:** Current treatments for AD

Drug	Approval	Prescription	Mode of Action	Clinical Efficacy	Adverse Effects	References
Tacrine	1993; withdrawn in 2013	application: orally dosage: 40–160 mg/day	noncompetitive, reversible inhibition of AChE and BChE	improving cognitive symptoms	hepatotoxicity	252, 253, 254
Donepezil	1996	application: orally dosage: 5–23 mg/day	reversible inhibition of AChE, increasing nAChR levels	improving cognitive and behavioral symptoms	well-tolerated, gastrointestinal effects	^256^^,^^257^^,^^267^^,^^284^
Rivastigmine	1997	application: orally or transdermally dosage: 3–12 mg/day (oral) or 4.6–9.5 mg/day (transdermal)	noncompetitive, pseudo-irreversible inhibition of AChE and BChE	reducing A*β* plaques, decreasing brain atrophy	well-tolerated, gastrointestinal effects	^254^^,^^266^^,^^284^^,^^285^
Galantamine	2001	application: orally dosage: 8-24 mg/day	competitive, reversible inhibition of AChE, opening of nAChR	reducing cognitive decline and mortality	well-tolerated, gastrointestinal effects	^254^^,^^258^^,^^259^^,^^262^^,^^284^
Memantine	2003	application: orally dosage: 5–28 mg/day	noncompetitive NMDAR antagonist, 5-HT3R antagonist	improving cognitive and behavioral symptoms	headache, dizziness, and gastrointestinal effects	^270^^,^^272^, ^273^, ^274^^,^^286^
Aducanumab	2021 not in Europe	application: intravenously dosage: 10 mg/kg	monoclonal antibody against soluble and insoluble A*β* aggregates	reducing A*β* plaques, slowing disease progression	amyloid-related imaging abnormalities	^276^^,^^279^
Lecanemab	2023 not in Europe	application: intravenously dosage: 10 mg/kg	monoclonal antibody against soluble and insoluble A*β* aggregates	reducing A*β* plaques and pTau levels, slowing disease progression	amyloid-related imaging abnormalities	^277^^,^^280^^,^^281^
Donanemab	2024 not in Europe	application: intravenously dosage: 700–1400 mg	monoclonal antibody against insoluble, modified, N-terminal truncated A*β*	reducing A*β* plaques and pTau levels, slowing disease progression	amyloid-related imaging abnormalities	^278^^,^^282^^,^^287^^,^^288^

Antagonists of the N-methyl-D-aspartate receptor (NMDAR) are another important group of AD drugs. Memantine reduces the NMDAR overactivation observed in patients with AD by blocking the NMDAR,^254^^,^^270^^,^^271^ which reduces A*β*-induced toxicity in vitro and improves disease progression in patients.^272^, ^273^, ^274^, ^275^

A novel group of AD therapeutics are antibodies targeting pathological protein aggregates. The human monoclonal antibodies aducanumab, lecanemab, and donanemab are approved for the treatment of AD with mild cognitive impairment or mild dementia. They target soluble and insoluble forms of pathological A*β*, resulting in reduced A*β* levels in the brain of patients with AD and decreased levels of phosphorylated tau in the CSF and blood.^276^, ^277^, ^278^, ^279^, ^280^, ^281^ While treatment with lecanemab and donanemab improves cognitive decline,^277^^,^^278^^,^^280^, ^281^, ^282^ the efficacy of aducanumab treatment on cognition has been inconsistent in clinical trials.^276^^,^^279^ The most common side effects of these antibodies were amyloid-related imaging abnormalities, including cerebral edema, effusions, and microhemorrhages.^276^, ^277^, ^278^, ^279^, ^280^, ^281^, ^282^

In addition to the above-mentioned drugs, behavioral and psychological symptoms of dementia, including psychotic, aggressive, depressive, and anxious behavior, can be treated with antipsychotics and antidepressants.^283^

### D Strategies targeting the serotonergic system

Currently, available drugs for dementia patients that target the serotonergic system include antipsychotics and antidepressants.^283^ However, a growing body of evidence suggests that targeted modulation of serotonergic neurotransmission has direct disease-modifying effects (Fig. 4). At the cellular level, these include neurotransmitter release, synaptic function, APP processing, protein aggregation, neuroinflammation, and neurodegeneration. In addition, both cognitive deficits and neuropsychiatric symptoms are improved by modulating the serotonergic system. Recent research has focused on SSRIs, MAOIs, and 5-HTR ligands (Table 3).^297^, ^298^, ^299^, ^300^, ^301^, ^302^, ^303^, ^304^, ^305^, ^306^, ^307^, ^308^, ^309^, ^310^, ^311^, ^312^, ^313^, ^314^, ^315^, ^316^, ^317^, ^318^, ^319^, ^320^, ^321^, ^322^, ^323^, ^324^, ^325^, ^326^, ^327^, ^328^, ^329^, ^330^, ^331^, ^332^, ^333^, ^334^, ^335^, ^336^, ^337^, ^338^, ^339^, ^340^, ^341^, ^342^, ^343^, ^344^, ^345^, ^346^, ^347^, ^348^, ^349^, ^350^, ^351^, ^352^, ^353^, ^354^, ^355^, ^356^, ^357^, ^358^, ^359^, ^360^, ^361^, ^362^, ^363^, ^364^, ^365^, ^366^, ^367^, ^368^, ^369^, ^370^, ^371^, ^372^, ^373^, ^374^, ^375^, ^376^, ^377^, ^378^, ^379^, ^380^, ^381^, ^382^, ^383^, ^384^, ^385^, ^386^, ^387^, ^388^, ^389^, ^390^, ^391^, ^392^, ^393^, ^394^, ^395^, ^396^, ^397^, ^398^, ^399^, ^400^, ^401^, ^402^, ^403^, ^404^, ^405^, ^406^, ^407^, ^408^, ^409^, ^410^, ^411^, ^412^, ^413^, ^414^, ^415^, ^416^, ^417^, ^418^, ^419^, ^420^, ^421^, ^422^, ^423^, ^424^ Preclinical validation of these approaches has been performed using several animal models that mimic the phenotype of AD and other dementias. Transgenic animal models mainly express mutant proteins involved in A*β* or tau aggregation, including *APP*, *PSEN1*, or *MAPT*. Phenotypically, most genetic animal models show amyloid deposits and cognitive decline. Other animal models use AChR or NMDAR antagonists to induce cholinergic or glutamatergic deficits that are characteristic of AD and cause cognitive impairment.

**Table 3:** Proposed serotonergic interventions in AD

Targeted Pathomechanisms/Symptoms	Action on the Serotonergic System	Drug	References
A*β* pathology	SSRI	Citalopram	297, 298, 299, 300
		S-citalopram	301, 302, 303
		Paroxetine	^304^
		Fluoxetine	^305^
	5-HT1AR antagonist	NAD-299	^306^
	5-HT2AR antagonist	Desloratadine	^307^
	5-HT2AR agonist	TCB-2	^306^
	5-HT2AR agonist + 5-HT1AR antagonist	TCB-2+NAD-299	^306^
	5-HT2CR agonist	Dexnorfenfluramine	^308^
	5-HT3R antagonist	Tropisetron	^309^
	5-HT4R agonist	RS-67333	310, 311, 312, 313
		ML-10302	^314^
		SSP-002392	^315^
	5-HT6R agonist	ST-1936	^314^
	5-HT7R agonist	AS-19	^314^
Tau pathology	SSRI	Citalopram	^299^
		S-citalopram	^302^^,^^316^, ^317^, ^318^, ^319^
		Paroxetine	^304^
		Trazodone	^320^
	5-HT7R inverse agonists/antagonist	SB-269970	^72^
		Amisulpride	^321^
		Lurasidone	^321^
		Mianserin	^321^
		Clozapine	^321^
APP metabolism	SSRI	Citalopram	^298^
		S-citalopram	^301^^,^^302^
	MAOI	Rasagiline	^322^
		TV-3326	^322^^,^^323^
	5-HT2CR agonist	Dexnorfenfluramine	^308^
	5-HT3R antagonist	Tropisetron	^309^
	5-HT4R agonist	RS-67333	^312^^,^^313^
		VRX-03011	^324^
		ML-10302	^314^^,^^325^
		SSP-002392	^315^
		Prucalopride	325, 326, 327
		Renzapride	^327^
		Usmarapride	^328^
	5-HT6R agonist	ST-1936	^314^
	5-HT7R agonist	AS-19	^314^
Neuroinflammation/oxidative stress/ROS production	SSRI	S-citalopram	^302^
	MAOI	Rasagiline	^329^
		TV-3326	^330^
	5-HT1AR antagonist	NAD-299	^331^
	5-HT2AR antagonist	Desloratadine	^307^
	5-HT2AR agonist	TCB-2	^331^
	5-HT2AR agonist + 5-HT1AR antagonist	TCB-2 + NAD-299	^331^
	5-HT3R antagonist	Y-25130	^332^
		MDL-72222	^332^
		Tropisetron	^333^
	5-HT4R agonist	RS-67333	^310^^,^^312^
Mitochondrial dysfunction	SSRI	Citalopram	^298^^,^^299^^,^^334^
	MAOI	Rasagiline	^323^
		TV-3326	^323^^,^^335^
BDNF	5-HT1AR antagonist	NAD-299	^306^
	5-HT2AR agonist	TCB-2	^306^
	5-HT2AR agonist + 5-HT1AR antagonist	TCB-2 + NAD-299	^306^
	5-HT6R antagonist	SB-742457	^336^
	5-HT6R agonist	WAY-181187	^336^
		WAY-208466	^337^
	5-HT7R agonist	LP-12	^338^
Neurotoxicity/apoptosis/neuronal loss/cell death	SSRI	Citalopram	^299^
		S-citalopram	^302^
		Trazodone	^320^
	MAOI	Rasagiline	^322^^,^^329^
		TV-3326	^322^^,^^323^^,^^330^^,^^335^^,^^339^
	5-HT1AR antagonist	NAD-299	^306^^,^^331^
	5-HT2AR agonist	TCB-2	^306^^,^^331^
	5-HT2AR agonist + 5-HT1AR antagonist	TCB-2 + NAD-299	^306^^,^^331^
	5-HT3R antagonist	Y-25130	^332^
		MDL-72222	^332^
		Tropisetron	^333^
	5-HT4R agonist	RS-67333	^311^
	5-HT7R inverse agonist/antagonist	SB-269970	^72^
	5-HT7R agonist	LP-211	^338^^,^^340^
		AS-19	^341^
Neuronal plasticity	SSRI	Citalopram	^299^^,^^334^
		S-citalopram	^318^^,^^319^
		Fluoxetine	^305^
	MAOI	Rasagiline	^329^
	5-HT2AR antagonist	Desloratadine	^307^
	5-HT4R agonist	Capeserod	^342^
	5-HT6R antagonist	SB-271046	^343^
	5-HT6R agonist	WAY-208466	^337^
	5-HT7R agonist	AS–19	^341^
Autophagy	5-HT2AR antagonist	Desloratadine	^307^
Calcium	5-HT3R antagonist	Y-25130	^332^
		MDL-72222	^332^
Cholinergic deficits	5-HT1AR antagonist	Lecozotan	^344^
	5-HT3R antagonist	Tropisetron	^345^
		Ondansetron	^345^
	5-HT4R agonist	VRX-03011	^324^
		BIMU-1	^346^
		BIMU-8	^346^
		Usmarapride	^328^
	5-HT6R antagonist	SB-357134	^347^
		SB-399885	^348^
		Pyrrolidinyl derivative “11”	^349^
	5-HT3R/5HT7R/5-HT1DR antagonist, 5-HT1AR/5-HT1BR agonist, SERT inhibitor	Vortioxetine	^350^
Glutamatergic deficits	5-HT1AR antagonist	WAY-100135	^351^
		NAN-190	^352^
		Lecozotan	^344^
	5-HT3R antagonist	Y-25130	^332^
		MDL-72222	^332^
	5-HT6R antagonist	SB-271046	^353^^,^^354^
		SB-357134	^347^
		Ro-4368554	^355^
Serotonergic deficits	5-HT1AR/5-HT7R antagonist	Arylpiperazine derivatives (HBK-14, HBK-15)	^356^
Excitability	5-HT4R agonist	Prucalopride	^357^
		Capeserod	^342^^,^^357^
		Zacopride	^358^
Cognitive impairments	SSRI	Citalopram	^334^
		S-citalopram	^302^^,^^316^
		Paroxetine	^304^
		Fluoxetine	^305^^,^^359^
	MAOI	Rasagiline	^322^^,^^329^
		TV-3326	^322^^,^^330^^,^^339^
	5-HT1AR antagonist	WAY-100635	360, 361, 362
		S-WAY-100135	^363^
		NAD-299	^306^
		NAN-190	^364^
		Lecozotan	^344^^,^^365^
		S-15535	^366^
	5-HT1AR agonist	8-OH-DPAT	^367^
		S-15535	^366^
	5-HT2AR antagonist	EMD-281014	^368^
		MDL-100907	^369^
		Desloratadine	^307^
	5-HT2AR agonist	TCB-2	^306^
	5-HT2AR agonist + 5-HT1AR antagonist	TCB-2 + NAD-299	^306^
	5-HT2CR antagonist	SB-242084	^370^
	5-HT2A/CR antagonist	Ritanserin	^360^^,^^371^
	5-HT3R antagonist	WAY-100579	^372^
		SEC-579	^373^
		Y-25130	^374^
		Ondansetron	^360^^,^^372^^,^^373^^,^^375^, ^376^, ^377^
		Itasetron	^378^
		Tropisetron	^309^^,^^333^^,^^379^
	5-HT4R agonist	RS-67333	^310^^,^^312^^,^^326^^,^^380^, ^381^, ^382^, ^383^, ^384^
		RS-17017	^385^
		BIMU-1	^384^^,^^386^, ^387^, ^388^
		BIMU-8	^386^
		VRX-03011	^324^
		Capeserod	^389^^,^^390^
		Prucalopride	^326^
		Usmarapride	^328^
	5-HT6R antagonist	SB-271046	^343^^,^^391^, ^392^, ^393^
		SB-357134	^394^
		SB-399885	^348^^,^^394^
		SB-742457	^336^^,^^395^^,^^396^
		Ro-04-6790	397, 398, 399
		Ro-4368554	^355^^,^^400^
		CMP X	^396^
		CMP Y	^396^
	5-HT6R agonist	WAY-181187	^336^
		WAY-208466	^337^
		EMD-386088	^393^
		E-6801	^393^
	5-HT7R inverse agonists/antagonist	SB-269970	401, 402, 403, 404
		Lurasidone	^402^^,^^405^^,^^406^
		Amisulpride	^321^^,^^402^
		Lurasidone + SB-269970	^402^
		Amisulpride + SB-269970	^402^
		Sulpiride + SB-269970	^402^
		Lurasidone + amisulpride	^402^
		Arylsulfonamide derivatives	^407^
	5-HT1AR/5-HT7R antagonist	Arylpiperazine derivative (HBK-15)	^356^
	5-HT3R/5HT7R/5-HT1DR antagonist, 5-HT1AR/5-HT1BR agonist, SERT inhibitor	Vortioxetine	^350^^,^^408^
	5-HT7R agonist	LP-211	^340^
		8-OH-DPAT	^409^
		AS-19	^410^
Behavioral and psychological symptoms of dementia (BPSD), including psychosis, depression, and anxiety	MAOI	TV-3326	^322^^,^^339^^,^^411^^,^^412^
	SSRI	Citalopram	^413^^,^^414^
		Sertraline	415, 416, 417, 418
		Fluoxetine	^359^
	5-HT1AR agonist	Tandospirone	419, 420, 421
	5-HT2AR inverse agonist/antagonist	Pimavanserin	^291^
	5-HT7R inverse agonists/antagonist	SB-269970	^404^^,^^422^^,^^423^
		Arylsulfonamide derivatives	^407^^,^^424^
	5-HT1AR/5-HT7R antagonist	Arylpiperazine derivatives (HBK-14, HBK-15)	^356^

#### 1 Modulation of 5-HT levels

To compensate for the lack of 5-HT observed in the brain and CSF of patients with AD, therapeutic approaches include the use of SSRIs and MAOIs. While they are currently widely used to alleviate depressive symptoms in patients with dementia, several preclinical studies suggest that certain SSRIs, including citalopram, paroxetine, fluoxetine, and trazodone, as well as the MAOIs rasagiline and TV-3326, may have disease-modifying effects in AD and FTD.

One of the best-studied examples is citalopram and its even more potent enantiomer, S-citalopram. In different transgenic AD mouse models, treatment with citalopram significantly reduced A*β* levels in the brain and alleviated cognitive impairment.^297^^,^^300^^,^^334^ On the cellular level, citalopram was shown to induce synaptogenesis and cell survival by reducing protein aggregation and improving mitochondrial function.^298^^,^^299^ Similar effects have been reported for S-citalopram treatment in various rodent disease models.^301^^,^^302^^,^^316^^,^^318^ Importantly, citalopram and S-citalopram target different cellular mechanisms relevant to AD and FTD pathology. Both have been shown to decrease A*β* levels in the brain and the brain interstitial fluid (ISF) by increasing *α*-secretase and decreasing *β*- and *γ*-secretase cleavage, thereby promoting nonamyloidogenic APP processing.^297^^,^^302^ Mechanistically, this process may be regulated by 3 pathways, including protein kinase B (Akt)/glycogen synthase kinase 3 *β* (GSK-3*β*), ERK, and c-Jun N-terminal kinase (JNK) signaling.^302^ In addition to its effects on A*β* pathology, S-citalopram treatment reduced tau hyperphosphorylation, as shown in different cell and animal models.^316^, ^317^, ^318^, ^319^ The underlying mechanism involves the activation of the Akt/GSK-3*β* pathway via 5-HT1AR.^319^ Furthermore, S-citalopram protects from deficits in neurite outgrowth and spine density, and increases the expression of postsynaptic markers, such as postsynaptic density (PSD)-95 and PSD-93. Furthermore, hippocampal neurodegeneration was alleviated by increasing neurotrophic factors and decreasing synaptic dysfunction, oxidative stress, and neuroinflammation.^302^^,^^318^^,^^319^

Further preclinical studies indicate that, in addition to citalopram, other SSRIs also have positive effects on the progression of dementia. For example, prophylactic, daily treatment with paroxetine for 5 months in a transgenic mouse model of AD ameliorated A*β* and tau levels as well as memory impairment compared to untreated mice.^304^ Moreover, fluoxetine treatment alleviated learning and memory deficits in mice with AD by reducing soluble A*β* and preventing synaptic and dendritic loss.^305^ Trazodone, another SSRI, was shown to reduce tau oligomerization, confirming the disease-modifying effects of SSRIs in the pathogenesis of AD and FTD.^320^

Studies in humans further support the beneficial effects of SSRI treatment. A retrospective analysis of cognitively normal elderly individuals revealed reduced A*β* plaques in the brain of participants who received citalopram treatment for 5 years compared with untreated participants, as shown by PET imaging.^297^ In addition, a clinical trial in healthy participants aged 21–50 years confirmed a 38% reduction in A*β* concentration in the CSF upon short-term treatment with citalopram.^300^ Similar effects have been reported for an 8-week treatment with S-citalopram in cognitively healthy elderly participants compared with the placebo-treated control group.^303^ However, despite the promising results in cognitively healthy individuals, clinical studies of citalopram and other SSRIs in patients with dementia have shown only moderate success. Although treatment with the SSRIs citalopram and sertraline improved psychotic symptoms, behavioral disturbances, and depression in demented patients, SSRI treatment was not effective in restoring cognitive function.^413^^,^^414^^,^^416^, ^417^, ^418^^,^^425^ Similar results were also obtained by the combined treatment of sertraline and the AChE inhibitor (AChEI) donepezil.^415^ Of note, one study found sex-specific differences in sertraline treatment: while cognitive performance in men worsened after drug administration, it improved in women.^417^ In contrast, the SSRI fluoxetine ameliorated both cognition and depression in AD patients with major depression.^359^ However, this study included only a small number of participants and was not placebo-controlled. In addition, it remains unclear whether the procognitive effects are a drug-specific effect or a result of the antidepressant action.

Another therapeutic approach to compensate for 5-HT deficiencies is to block 5-HT degradation using MAOIs. The selective MAO-B inhibitor rasagiline improved cognitive function and elicited neuroprotective properties in aged mice by increasing synaptic plasticity, antiapoptotic and antioxidant proteins, while decreasing proapoptotic markers.^329^ Furthermore, the brain-selective MAOI and ChE inhibitor TV-3326 ameliorated cognitive deficits in rats induced by the mAChR antagonist scopolamine and in aged rhesus monkeys.^339^^,^^411^ The beneficial effects on cognition may be due to its neuroprotective properties, including prevention of the mitochondrial membrane potential disruption and caspase-3-mediated cell death by increasing antiapoptotic and decreasing proapoptotic markers.^322^^,^^323^^,^^330^^,^^335^^,^^339^ Furthermore, TV-3326 reduced full-length APP and simultaneously promoted the secretion of the nonamyloidogenic sAPP*α*, potentially by enhancing *α*-secretase activity mediated by protein kinase C/mitogen-activated protein kinase signaling in vitro.^323^ However, the antiapoptotic and neuroprotective effects as well as the modulation of APP processing of rasagiline and TV-3326 seem to be independent of MAO inhibition, at least in cell culture.^323^^,^^339^^,^^426^ In addition, TV-3326 showed antidepressant and anxiolytic properties in rats.^339^^,^^412^ However, despite these promising preclinical results, the selective MAO inhibitor selegiline did not improve cognitive behavior, activities of daily living, or emotional state in clinical studies with AD patients.^427^^,^^428^

#### 2 Targeting the 5-HT1R

Postsynaptic 5-HT1ARs are highly expressed in cortical and hippocampal brain regions, suggesting a crucial role in learning and memory.^429^, ^430^, ^431^, ^432^ Moreover, 5-HT1Rs can modulate the cholinergic and glutamatergic systems that are involved in cognitive function.^344^^,^^351^^,^^352^^,^^433^^,^^434^ Targeting the 5-HT1AR with antagonists has been proven to restore neurotransmitter deficiencies accompanied by amelioration of cognitive impairment in animal models. Particularly, the 5-HT1AR antagonists WAY-100135, NAN-190, and lecozotan increased acetylcholine and glutamate release in rodents.^344^^,^^351^^,^^352^ In line with this, selective 5-HT1AR blockade with NAD-299 had neuroprotective effects by reducing brain A*β* deposition, hippocampal neuronal loss, and oxidative stress, while simultaneously increasing hippocampal levels of brain-derived neurotrophic factor (BDNF) in an AD rat model.^306^^,^^331^ Consequently, numerous 5-HT1AR antagonists, such as WAY-100635, S-WAY-100135, NAN-190, NAD-299, and lecozotan, improved learning and memory in wild-type (WT) animals and rescued cognitive impairment induced by aging, cholinergic, glutamatergic, or serotonergic deficits in rodent models, as well as in marmosets and rhesus monkeys.^344^^,^^360^, ^361^, ^362^, ^363^, ^364^, ^365^, ^366^^,^^435^, ^436^, ^437^, ^438^, ^439^

Although blocking the 5-HT1AR may be a promising approach to modify the progression of dementia, some studies reported that activating the 5-HT1AR may also have procognitive effects. 5-HT1AR agonists enhance cognitive performance in mice and rescue scopolamine-induced impairments in spatial learning in rats.^366^^,^^367^^,^^437^^,^^440^^,^^441^ In patients with AD, the 5-HT1AR partial agonist tandospirone ameliorated anxiety and depressive symptoms as well as behavioral and psychological symptoms.^419^, ^420^, ^421^ However, the 5-HT1R agonist xaliproden has failed to show any therapeutic efficacy in clinical settings (NCT00104013, NCT00103649).

The contradictory findings that both agonists and antagonists might improve cognitive function could be explained by regional selectivity and different doses of the ligands used. Low doses of agonists may have beneficial effects on learning and memory by targeting presynaptic 5-HT1ARs, while the cognitive-enhancing properties of antagonists may result from blocking postsynaptic 5-HT1ARs, thereby enhancing cholinergic and glutamatergic transmission. ^363^^,^^366^^,^^437^^,^^440^, ^441^, ^442^

#### 3 Targeting the 5-HT2R

As mentioned in section *III B*, polymorphisms in the *HTR2* gene impact A*β* levels in the CSF and are associated with various neuropsychiatric symptoms in AD, suggesting a crucial role of 5-HT2R in dementia.^218^^,^^222^, ^223^, ^224^, ^225^, ^226^, ^227^ Several studies provide evidence that blocking 5-HT2AR by specific antagonists may improve disease progression and ameliorate dementia-associated symptoms, such as psychosis. In particular, the antiallergic drug desloratadine (DLT), with antagonistic effects on the 5-HT2AR, ameliorated cognitive deficits in APP/PS1 mice by promoting hippocampal synaptic plasticity and integrity.^307^ The cognitive-enhancing effects of 5-HT2AR antagonists have also been confirmed in rodents as well as in nonhuman primates, highlighting the critical role of 5-HT2AR in cognition.^368^^,^^369^^,^^371^ These procognitive effects may result from GABAergic disinhibition. Because the activation of 5-HT2R can increase GABAergic neurotransmission and reduce ACh release in the hippocampus, antagonizing the 5-HT2R in GABAergic neurons may increase ACh release by blocking GABAergic inhibition of the cholinergic system.^443^^,^^444^ In this context, an ongoing phase 2 clinical trial is investigating the effect of 5-HT2AR antagonist trazodone on sleep, hippocampal-dependent memory, and hippocampal excitability (NCT05282550).

In addition to their beneficial effects on cognition, 5-HT2AR antagonists may also influence microglial function. DLT treatment has been shown to promote phagocytosis of A*β* by upregulating the phagocytic toll-like receptor 2 and 4 in microglia. In turn, this DLT-mediated upregulation has been reported to result from the 5-HT2AR/cAMP/PKA/CREB/glucocorticoid receptor pathway. DLT also improved microglial A*β* clearance by stimulating 5-HT2AR-mediated autophagy and reduced neuroinflammation, which was mediated by 5-HT2AR/cAMP/PKA/CREB/Sirtuin 1 signaling.^307^

Modulation of the 5-HT2AR activity is also a promising strategy for targeting neuropsychiatric symptoms in dementia. Pimavanserin, a selective 5-HT2AR inverse agonist and antagonist, has been evaluated in a phase 3 discontinuation trial with patients suffering from dementia-related psychosis. After an initial treatment phase of 12 weeks, patients who responded to pimavanserin treatment were randomized in a double-blind fashion to continue pimavanserin treatment or to switch to a placebo. After 26 weeks, pimavanserin-treated patients had significantly fewer psychotic relapses compared to the placebo control, indicating that the risk of relapse decreased after receiving pimavanserin.^291^ In addition, a current phase 2 or 3 clinical trial is evaluating the efficacy of the 5-HT2A inverse agonist ACP-204 in patients with AD affected by psychosis (NCT06159673).

In contrast, several studies demonstrated that activating the 5-HT2AR may also prevent disease progression. Treatment with the 5-HT2AR agonist TCB-2 and in combination with the 5-HT1AR antagonist NAD-299 improved cognitive performance in WT mice and a rat model of AD.^306^^,^^445^ The observed beneficial effect is mediated by reducing A*β* plaques, oxidative stress, and neuronal loss in the hippocampus while simultaneously increasing BDNF levels, which has neuroprotective effects.^306^^,^^331^

Some studies also suggest the 5-HT2CR as a potential therapeutic target in AD. However, the results are still controversial. While blocking the 5-HT2CR with the antagonist SB-242084 exhibited an amelioration in spatial reversal learning compared with vehicle-treated WT mice,^370^ activation of the 5-HT2CR with the agonist dexnorfenfluramine promoted the production of the neuroprotective sAPP*α* and attenuated A*β* levels in the CSF of guinea pigs.^308^

#### 4 Targeting the 5-HT3R

Accumulating studies provide evidence that blocking the 5-HT3R activity by specific receptor antagonists may improve different pathophysiological phenotypes in dementia. In rat cortical neurons, the 5-HT3R antagonists Y-25130 and MDL-72222 have been shown to protect against A*β*-induced neurotoxicity by decreasing neuronal cell death, calcium increase, glutamate release, and ROS production.^332^ In line with this, treatment with tropisetron, a clinically approved antiemetic drug with a potent 5-HT3R antagonism, protected rats from A*β*-induced neurotoxicity.^333^ Besides its antagonism toward the 5-HT3R, tropisetron also binds to the APP ectodomain with a lower affinity. It has been shown to enhance sAPP*α* levels while decreasing A*β* loads in neurons of a transgenic AD mouse model.^309^ Interestingly, tropisetron and ondansetron, another antiemetic drug with 5-HT3R antagonism, inhibited the 5-HT-mediated blockade of ACh release in the human cerebral cortex, thereby compensating for the impaired ACh neurotransmission observed in patients with AD and FTD.^345^ In line with this, several studies demonstrated beneficial effects of 5-HT3R antagonists, including WAY-100579, Y-25130, SEC-579, tropisetron, ondansetron, and itasetron, on cognition both in aged animals and AD models.^309^^,^^333^^,^^360^^,^^372^, ^373^, ^374^, ^375^, ^376^, ^377^, ^378^, ^379^

#### 5 Targeting the 5-HT4R

Because of the important role of 5-HT4R in learning and memory, activation of this receptor by agonists has been proposed as a potential therapeutic approach for the treatment of dementia.

Several 5-HT4R agonists, including RS-67333, VRX-03011, ML-10302, SSP-002392, prucalopride, renzapride, and usmarapride, are neuroprotective by increasing nonamyloidogenic sAPP*α* levels in vitro and in the cortex, hippocampus, and CSF of both WT rodents and several transgenic mouse models of AD.^312^^,^^313^^,^^315^^,^^324^^,^^325^^,^^327^^,^^328^ One underlying mechanism may involve 5-HT4R-mediated activation of the cAMP/Rap guanine nucleotide exchange factor 3 (Epac1)/Rap GTP-binding protein 1 (Rap1)/Rac pathway leading to sAPP*α* secretion.^446^ Another possible mechanism is based on the 5-HT4R-mediated expression of the matrix metalloproteinase 9 (MMP-9), which promotes *α*-secretase-like APP cleavage. This was demonstrated both in vitro and in a transgenic mouse model of AD.^313^ Combined treatment of the selective 5-HT4R agonist prucalopride and the AChEI donepezil, each in an ineffective low dose, further increased the sAPP*α* levels compared with prucalopride treatment alone.^325^ In line with this, 5-HT4R activation by RS-67333, ML-10302, or SSP-002392 attenuated A*β* pathology, neuroinflammation, and neuronal loss in multiple transgenic AD mouse models.^310^, ^311^, ^312^, ^313^, ^314^, ^315^ Mechanistically, A*β* reduction in the ISF may be explained by PKA-mediated activation of the mitogen-activated protein kinase kinase (MEK)/ERK cascade. The authors further speculated that ERK may enhance *α*-secretase cleavage, thus attenuating A*β* production.^314^ However, another study demonstrated a decrease in APP and *β*-secretase *β*-site amyloid precursor protein cleaving enzyme 1 expression rather than an activation of *α*-secretase.^315^

In addition to facilitating nonamyloidogenic APP cleavage, 5-HT4R activation has beneficial effects on neuronal excitability and spine growth in mice^342^^,^^357^^,^^358^ and ACh release in rats.^324^^,^^328^^,^^346^ Consequently, 5-HT4R activation by numerous selective and partial agonists, such as RS-67333, RS-17017, VRX-03011, BIMU-1, BIMU-8, capeserod, prucalopride, and usmarapride, has been reported to improve cognitive performance, including spatial learning as well as recognition, working, episodic, social, emotional, and associative memory. This has been validated in younger and older macaques, as well as in WT rodents and multiple rodent models with cognitive deficits induced by either aging, mAChR antagonists, atropine, the NMDAR antagonist MK-801, 5-HT4R antagonists, or genetic modifications.^310^^,^^312^^,^^325^^,^^326^^,^^328^^,^^380^^,^^383^, ^384^, ^385^, ^386^, ^387^, ^388^, ^389^, ^390^^,^^447^ Synergistic treatment of 5-HT4R agonists and AChEIs, each at noneffective doses, also improved cognitive function while reducing side effects of the individual compounds.^381^^,^^382^^,^^390^

Although preclinical data from in vitro experiments and animal studies have provided compelling evidence that 5-HT4R activation may have neuroprotective effects, clinical studies have been less promising so far. The 5-HT4R agonists PRX-03140 and PF-04995274 were already evaluated in phase 1 and 2 clinical trials. While studies evaluating its impact on learning and memory have been discontinued (NCT00672945, NCT01345864), a phase 1 trial investigating sAPP*α* levels in the CSF upon PF-04995274 treatment has been completed (NCT01193062). However, no results have been published yet.

#### 6 Targeting the 5-HT6R

The 5-HT6R is a known regulator of several neurotransmitter systems, including ACh, glutamate, and GABA,^347^^,^^349^^,^^448^^,^^449^ and has therefore been suggested as a possible target in AD. As it is almost exclusively expressed in the CNS, targeting this receptor may avoid peripheral side effects.^450^^,^^451^ However, because both agonists and antagonists may have disease-modifying effects, the role of 5-HT6R as a potential target in AD warrants further investigation.

Blocking the 5-HT6R has been shown to enhance acetylcholine levels.^347^, ^348^, ^349^ In line with this, cognitive impairment in rats caused by scopolamine-induced cholinergic deficits was restored by numerous 5-HT6R antagonists, including SB-271046, SB-399885, SB-742457, Ro-04-6790, Ro-4368554, CMP X, and CMP Y.^348^^,^^355^^,^^391^^,^^393^^,^^396^^,^^398^, ^399^, ^400^ In contrast, another study reported that the 5-HT6R antagonist SB-271046 reversed scopolamine-induced learning deficits in rats only when applied in combination with the AChEI galanthamine.^392^

The cholinergic system is not the only one modulated by 5-HT6R antagonists. SB-271046 has been shown to increase glutamate levels in the frontal cortex and dorsal hippocampus of rats.^353^^,^^354^ Inducing glutamatergic deficits by administration of the NMDAR antagonist MK-801 results in impaired cognitive performance in rodents, which can be rescued by treating these animals with the 5-HT6R antagonists SB-271046 or SB-742457.^336^^,^^392^^,^^393^ In addition to monotherapy, treatment of SB-271046 in combination with the AChEI galanthamine synergistically rescued learning deficits in the same animal model.^392^ The impact of the 5-HT6R on the glutamatergic system was further confirmed by a study showing that the procognitive effects induced by the 5-HT6R antagonist Ro-04-6790 were blocked by the NMDAR antagonist MK-801, suggesting an increase in glutamatergic neurotransmission as a potential mechanism of action.^397^ In addition to neurotransmitter-based animal models, antagonists of the 5-HT6R also improved cognitive performance in untreated adult rats, cognitively impaired aged rats, and a transgenic AD mouse model.^343^^,^^355^^,^^391^^,^^394^, ^395^, ^396^ Blocking the 5-HT6R in the AD mouse model rescued defects in neuronal primary cilia morphology, suggesting that the 5-HT6R is involved in regulating neuronal morphology to improve cognitive function.^343^

While blocking the 5-HT6R seems to be beneficial by rescuing both cholinergic and glutamatergic deficits, there is evidence that activation of the 5-HT6R affects the GABAergic system. Several studies have reported an increase in GABA release in rat CA1 neurons and several brain regions, including frontal cortex, dorsal hippocampus, striatum, and amygdala, after treatment with the selective agonist WAY-181187.^448^^,^^452^ In addition to increased GABA levels, West et al^452^ observed decreased long-term potentiation (LTP) and speculated that the 5-HT6R-mediated increase in GABAergic neurotransmission may contribute to LTP attenuation. However, several 5-HT6R agonists, such as WAY-181187, WAY-208466, EMD-386088, and E-6801, improved cognitive performance, including short- and long-term memory and associative and emotional learning, in WT as well as in cognitively impaired rodents induced by mAChR or NMDAR antagonists.^336^^,^^337^^,^^393^ The impact on learning was mediated by increasing neuronal plasticity and BDNF levels, potentially via MEK/ERK signaling.^336^^,^^337^ There are several hypotheses to explain why both 5-HT6R agonists and antagonists have beneficial effects on cognition. One is based on regional selectivity: while agonists activate 5-HT6Rs located on cholinergic and glutamatergic neurons, thereby enhancing cholinergic and glutamatergic neurotransmission, antagonists are predicted to stimulate 5-HT6Rs expressed on GABAergic interneurons, resulting in GABAergic disinhibition and thus also increasing cholinergic and glutamatergic activity. Another hypothesis is based on the functional selectivity of the ligands, suggesting that agonists stimulate different pathways than antagonists block. Moreover, the ligands used may not be selective for the 5-HT6R and may also act on other proteins responsible for procognitive effects.^453^

Importantly, activation of 5-HT6R not only improved cognition but also reduced A*β* levels in the ISF of transgenic AD mice via PKA/MEK/ERK, signaling as already described for the 5-HT4R.^314^ Despite the promising effects of 5-HT6R activation in preclinical studies, 5-HT6R antagonists have been the drugs of choice in clinical trials for AD. However, all of them have failed so far in phase 2 or 3 clinical trials because of lack of efficacy.^454^

#### 7 Targeting the 5-HT7R

The 5-HT7R emerged as an interesting target in dementia because of its broad distribution in the CNS, especially in the hippocampus, cortex, and thalamus, regions primarily affected in AD and FTD.^432^^,^^455^^,^^456^

In particular, blocking the 5-HT7R has been suggested to have disease-modifying effects by improving both tau pathology and cognition. We have recently demonstrated that the inverse agonist SB-269970, which specifically blocks the constitutive activity of 5-HT7R, reduces pathological tau hyperphosphorylation and aggregation, and alleviates toxicity in neural cells induced by overexpression of an FTD-associated tau mutant.^72^ In line with this, SB-269970 and other selective and nonselective 5-HT7R antagonists, such as arylsulfonamide derivatives and HBK-15, have been shown to enhance learning and memory in WT rodents and attenuate cognitive deficits in dementia animal models.^356^^,^^401^, ^402^, ^403^, ^404^^,^^407^

In addition to SB-269970, many clinically approved drugs that have a multimodal profile with binding affinities for various receptors also act as antagonists or inverse agonists at the 5-HT7R and may have beneficial effects in the treatment of dementia. For example, the antidepressant vortioxetine, which is a 5-HT7R antagonist, increased ACh release in the medial prefrontal cortex of rats and improved memory in both WT rats and 5-HT-depleted cognitively impaired rats.^350^^,^^408^ A recently initiated phase 2 clinical trial is investigating whether vortioxetine improves mood symptoms and cognitive function in patients with an early-stage behavioral variant of FTD (NCT06604520). The antipsychotic lurasidone acts as a 5-HT7R inverse agonist, which reduced 5-HT7R-mediated tau phosphorylation and aggregation in vitro,^321^ and attenuated cognitive deficits induced by NMDAR antagonists.^402^^,^^405^^,^^406^ Moreover, our recent study identified the antipsychotic amisulpride as another promising candidate. It has been shown to block the 5-HT7R constitutive activity by acting as an inverse agonist and improving tau pathology in various in vitro and in vivo models of tauopathy.^72^ In line with this, it ameliorated memory deficits in 2 FTD mouse models and in NMDAR-deficient rats.^321^^,^^402^^,^^457^ Interestingly, the chemically similar drug sulpiride, which is less potent in blocking the 5-HT7R, improved memory performance only in combination with the selective 5-HT7R inverse agonist SB-269970, highlighting a 5-HT7R-specific effect.^402^ In addition, combined treatments of the aforementioned 5-HT7R blockers are also promising in targeting cognitive deficits.^402^

Of note, some studies reported that the activation of 5-HT7R may also have cognitive-enhancing effects.^409^^,^^410^ Particularly, the specific 5-HT7R agonists AS-19 and LP-211 improved A*β-*, scopolamine-, and MK-801-induced memory deficits in rats.^340^^,^^410^ Additionally, stimulation of 5-HT7R with LP-12 had neuroprotective effects in vitro.^338^ In a rat model of AD, the 5-HT7R AS-19 increased hippocampal LTP and reduced neuronal loss.^341^ Furthermore, AS-19 attenuated ISF A*β* levels in a transgenic AD mouse model. This effect may be mediated by a PKA-induced activation of MEK/ERK signaling, similar to those shown for the 5-HT4R and 5-HT6R.^314^

Besides targeting disease-related mechanisms, modulation of the 5-HT7R may also be beneficial in treating dementia-associated symptoms, such as psychosis and depression. Blocking the 5-HT7R with antagonists or inverse agonists, including SB-269970, arylsulfonamide derivatives, HBK-14, and HBK-15, had antipsychotic and antidepressant effects in rodents.^356^^,^^404^^,^^407^^,^^422^, ^423^, ^424^

Taken together, these data clearly indicate that modulation of different components of the serotonergic system can counteract multiple cellular processes involved in the pathogenesis of AD and FTD, highlighting its promising potential not only for the treatment of dementia-associated neuropsychiatric symptoms, but also as a disease-modifying approach. So far, however, none of the drugs tested in clinical trials has been approved.

## IV Parkinson's disease

### A Disease-specific pathological phenotypes

PD is the second most common neurodegenerative disease, affecting an estimated 10 million people worldwide (Table 1). In contrast to AD, PD is disproportionately prevalent in men than in women.^88^^,^^89^ PD is characterized by a progressive loss of dopaminergic neurons in the nigrostriatal system, which, as a part of the basal ganglia, is critically involved in the regulation of motor function.^95^ Consequently, PD manifests as motor symptoms such as bradykinesia, rigidity, and tremor. In addition, PD is characterized by several nonmotor symptoms, including depression, cognitive decline, psychosis, pain, sleep disturbances, and gastrointestinal dysfunction.^94^ The onset of PD is assumed to be driven by a combination of various factors,^458^ with age being a major risk factor.^459^ In addition, genetic factors can dramatically increase susceptibility to the disease (Table 1).

Similar to other neurodegenerative diseases, pathological protein aggregation – in this case of the *α*-syn protein - is a hallmark of PD. Physiological *α*-syn regulates synaptic function and plasticity by interacting with the SNARE complex and synaptic vesicles.^460^^,^^461^ Abnormal deposition of misfolded *α*-syn within the cell body and neurites of neurons, known as Lewy bodies and Lewy neurites, contributes to synaptic dysfunction, impaired neurotransmission, decreased excitability, and neurotoxicity.^462^, ^463^, ^464^ *α*-Syn has high self-aggregating properties that can be triggered by mutations in the *SNCA* gene or dysfunctional protein degradation mechanisms.^465^, ^466^, ^467^, ^468^, ^469^ Posttranslational modifications, such as phosphorylation, ubiquitination, SUMOylation, nitration, O-GlcNacylation, and truncation, also promote its aggregation propensity.^470^ However, the exact pathomechanism, by which *α*-syn drives the progression of PD remains elusive. In addition to the characteristic *α*-syn aggregates, A*β* plaques, NFTs and TDP-43 inclusions have also been observed in patients with PD .^471^, ^472^, ^473^, ^474^, ^475^

Besides protein aggregation, other molecular mechanisms may contribute to the progressive loss of dopaminergic neurons and the development of clinical symptoms of PD. These include changes in calcium homeostasis, mitochondrial and lysosomal dysfunctions, impaired vesicle trafficking and synaptic function, oxidative stress, and neuroinflammation.^476^, ^477^, ^478^, ^479^, ^480^, ^481^, ^482^, ^483^, ^484^, ^485^, ^486^

### B Serotonergic imbalances

**Fig. 5:**
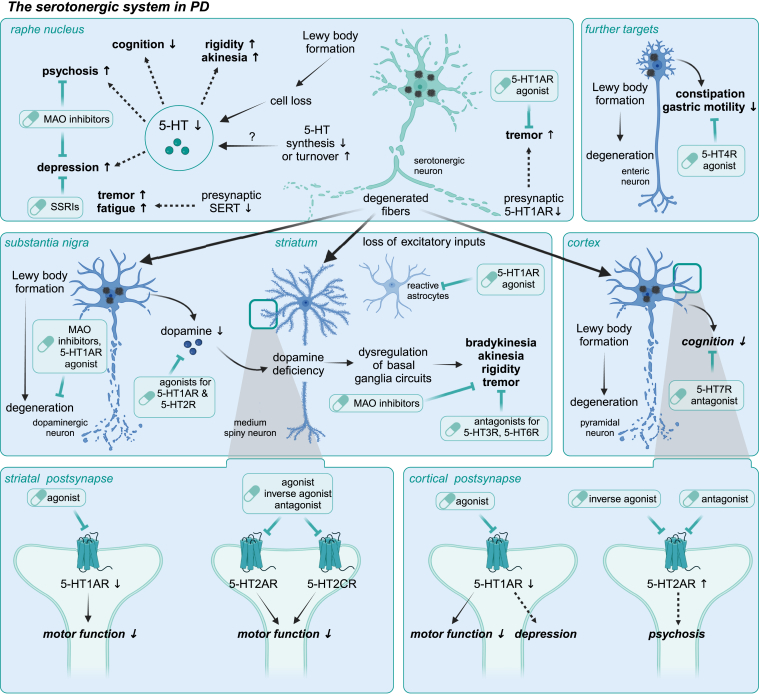
Serotonergic deficiencies in PD and its therapeutic targeting. Overview of serotonergic changes in the brain and gastrointestinal tract during the progression of PD. Early in the course of the disease, Lewy body formation drives the loss of serotonin-producing neurons in the raphe nucleus (upper left panel). In addition, impaired 5-HT synthesis and/or turnover may further contribute to reduced 5-HT levels. The collapse of serotonergic projections to the substantia nigra, striatum, and cortex, as well as reduced levels of presynaptic SERT and 5-HT1AR, are found in patients with PD. These serotonergic changes have been reported to correlate with motor and nonmotor symptoms of PD. In the substantia nigra, the Lewy body-driven degeneration of dopaminergic neurons leads to dopamine deficiency in striatal neurons, resulting in dysregulation of basal ganglia circuits (middle left panel). Loss of excitatory serotonergic input to these brain regions and imbalanced 5-HTR expression may further contribute to the dysregulation and thus the manifestation of motor symptoms in the early stages of PD (lower left panel). In later stages, degeneration of cortical neurons and altered postsynaptic 5-HTR levels are involved in the development of nonmotor symptoms in PD (middle and lower right panels). In addition, pathological protein aggregation in enteric neurons in the gastrointestinal tract also impacts the disease progression of PD. The potential direct consequences of serotonergic alterations are indicated by arrows with solid lines, and correlations by arrows with dashed lines. Proposed targeted interventions for serotonergic imbalances are highlighted by green boxes with a pill symbol.

The progressive loss of dopaminergic neurons and its associated motor symptoms, such as tremors and bradykinesia, are characteristic of PD. However, the occurrence of nonmotor symptoms suggests the involvement of other neurotransmitter systems in disease development. In particular, alterations in 5-HT neurotransmission are assumed to play a crucial role in PD (Fig. 5).

Serotonergic neurons project to numerous brain regions that degenerate in PD, including the substantia nigra, striatum, globus pallidus, subthalamic nucleus, thalamus, hypothalamus, and cortex.^487^, ^488^, ^489^, ^490^, ^491^, ^492^, ^493^ Several studies found loss of the 5-HT-producing neurons in the raphe nuclei of patients with PD.^494^, ^495^, ^496^ Lewy body formation has also been detected in the median and dorsal raphe nucleus, which may contribute to the neurodegeneration in this region. Notably, these pathological changes occur early in the course of PD, long before the substantia nigra in the midbrain is affected.^493^^,^^496^^,^^497^ The loss of neurons in the dorsal raphe nucleus is even more pronounced in patients with PD with depression than in nondepressed patients.^494^ Further serotonergic imbalances are related to presynaptic 5-HT1ARs, which are drastically reduced in the midbrain raphe nuclei and correlate with parkinsonian tremor.^498^

In line with neurodegeneration observed in the raphe nuclei, 5-HT levels are decreased by up to 85% in several brain regions affected in PD, including the substantia nigra, striatum, globus pallidus, hypothalamus, thalamus, cortex, and hippocampus.^192^^,^^499^, ^500^, ^501^, ^502^, ^503^ Furthermore, reduced 5-HT levels have been found in the CSF of patients with PD, which inversely correlate with the motor symptoms of rigidity and akinesia.^504^ In addition to 5-HT, CSF levels of its direct precursor 5-HTP are also attenuated in patients with PD.^504^^,^^505^ In contrast, the concentrations of the 5-HT preprecursor TRP are not altered in the CSF, indicating that the conversion of TRP to 5-HTP may be impaired.^504^^,^^505^ On the other hand, the activity of the rate-limiting enzyme TPH, which catalyzes this reaction, is significantly diminished only in the thalamus and not in other PD-affected brain regions, such as the substantia nigra, striatum, globus pallidus, hypothalamus, and raphe nuclei.^506^ Interestingly, oxidative stress leads to aggregation of the isoform TPH2, accompanied by decreased enzyme activity, which may contribute to serotonergic degeneration in PD.^507^ The levels of the 5-HT metabolite 5-HIAA in PD seem to vary depending on the brain region. While it is decreased in the dorsal striatum and hippocampus, it is not affected in the frontal, entorhinal, and cingulate cortex.^192^^,^^501^^,^^503^ Whether 5-HIAA concentration is reduced in the CSF remains unclear, as only a few studies have shown significant differences between the patients with PD and healthy controls.^508^, ^509^, ^510^, ^511^, ^512^, ^513^, ^514^, ^515^, ^516^ Studies in animal models of PD are not fully consistent with the observations in patients. In rodent and marmoset models of PD, induced either by dopaminergic lesioning or vesicular monoamine transporter 2 deficiency, decreased 5-HT levels were found in cortical regions, the hippocampus, and the striatum.^517^, ^518^, ^519^, ^520^, ^521^ This was partly accompanied by an increase in the 5-HIAA/5-HT ratio, indicating an elevated 5-HT turnover rather than impaired biosynthesis.^518^^,^^522^ In contrast, other studies reported no changes in 5-HT and its metabolites in these brain regions.^519^^,^^523^, ^524^, ^525^ Other reports have shown even increased 5-HT and 5-HIAA levels in the striatum,^517^^,^^526^, ^527^, ^528^, ^529^, ^530^, ^531^ and enhanced striatal 5-HT fiber innervation,^531^^,^^532^ indicating that the available animal models do not fully resemble the serotonergic deficiencies observed in patients with PD.

Multiple studies using SPECT, PET, and postmortem samples consistently reported reduced SERT densities in the striatum, thalamus, hypothalamus, basal ganglia, amygdala, midbrain, hippocampus, cortical regions, raphe nuclei, and brainstem of patients with PD at early and late disease stages.^192^^,^^500^^,^^533^, ^534^, ^535^, ^536^, ^537^, ^538^, ^539^, ^540^, ^541^ In contrast, a few studies revealed no changes in the midbrain and other brain regions in early-stage patients with PD.^542^^,^^543^ Generally, lower SERT levels negatively correlate with tremors and fatigue, as well as with mental activity behavior and mood in patients with PD.^534^^,^^537^^,^^544^ Furthermore, polymorphisms in the *SERT* gene have been implicated in PD pathogenesis and may contribute to depressive phenotypes in PD.^545^^,^^546^

Serotonergic neurotransmission in PD also seems to be affected at the postsynaptic level, as brain region-specific changes in the expression of some 5-HTRs have been reported in patients with PD. Lower postsynaptic 5-HT1AR levels in cortical regions, the ventral striatum, and the hippocampus have been observed using PET.^547^ Of note, PD patients with depression have lower 5-HT1AR levels than nondepressed patients with PD, suggesting a critical role of the 5-HT1AR in the development of depression in PD.^547^ 5-HT2AR levels are reduced in the temporal cortex of patients with PD, while they are enhanced in the neocortex.^548^, ^549^, ^550^ In patients with PD with visual hallucinations, 5-HT2AR levels are particularly increased in the visual cortex, temporal cortex, prefrontal cortex, and insula, compared with PD patients without these symptoms, suggesting that the 5-HT2AR is involved in the development of these nonmotor PD symptoms.^549^^,^^551^ Whether the expression levels of 5-HT3R, 5-HT4R, 5-HT5R, 5-HT6R, and 5-HT7R are altered during disease onset and progression warrants further investigation due to insufficient or inconsistent data.^228^^,^^552^, ^553^, ^554^, ^555^, ^556^, ^557^ However, genetic alterations in specific receptor genes have been implicated in PD. A variant in the *HTR2A* gene is associated with impulsive compulsive behavior in PD,^558^ while a polymorphism in the *HTR6* may be associated with a lower risk of developing PD.^559^

Imbalances in serotonergic neurotransmission commonly observed in patients with PD may contribute significantly to the pathological phenotypes. The basal ganglia are innervated by predominantly excitatory serotonergic inputs from the raphe nuclei.^560^ Raphe nuclei degeneration, in combination with dopamine depletion in the striatum and substantia nigra, directly affects the basal ganglia and may therefore be responsible for a net decrease in motor cortex activity leading to the characteristic parkinsonian symptoms (Fig. 5). Although Lewy body formation in the raphe begins early in the disease, long before the dopaminergic system becomes affected,^493^^,^^496^^,^^497^ the degeneration of serotonergic fibers appears to be much slower than that of dopaminergic neurons.^500^^,^^540^^,^^543^ However, the pronounced alterations in serotonergic neurotransmission that are observed in the late stages of the disease may be particularly responsible for the nonmotor symptoms, such as cognitive impairment and psycho-emotional disturbances.

### C Current treatment strategies

The most common strategy for treating the motor symptoms of PD is to compensate for dopaminergic deficits (Table 4).^563^, ^564^, ^565^, ^566^, ^567^, ^568^, ^569^, ^570^, ^571^, ^572^, ^573^, ^574^ This includes the application of the dopamine precursor levodopa (L-DOPA), which is usually administered in combination with the decarboxylase inhibitor carbidopa, and dopamine receptor (DR) agonists.^292^^,^^561^ In addition, MAO-B inhibitors, and catechol-O-methyltransferase inhibitors are used to slow down the degradation of released dopamine.^292^ Amantadine increases dopamine levels by antagonizing NMDARs, thereby improving treatment-induced dyskinesia and extrapyramidal side effects.^292^^,^^562^ In cases of severe tremor, anticholinergic drugs are often prescribed despite their potential adverse effects on cognitive deficits.^292^ Of note, long-term treatment with L-DOPA causes dyskinesia, or the so-called “on-off” phenomenon, based on fluctuations in drug response. More than 40% of patients with PD are affected by L-DOPA-induced dyskinesia 5 years after treatment initiation.^563^

**Table 4:** Current treatments for PD

Drug	Approval	Prescription	Mode of Action	Clinical Efficacy	Adverse Effects	References
Levodopa	1975	application: orally dosage: 75–435 mg/day	dopamine precursor	treating motor symptoms	levodopa-induced dyskinesia, on-off phenomenon, nausea	^563^^,^^567^^,^^568^
Pramipexole	2010	application: orally dosage: 0.375 mg/day	dopamine receptor agonist	treating motor symptoms	orthostatic hypotension, dizziness, nausea, sleepiness	^292^^,^^561^^,^^566^^,^^569^
Ropinirole	1997	application: orally dosage: 0.75–2 mg/day				
Rotigotine	2007	application: transdermally dosage: 2 mg/day				
Apomorphine	2004	application: subcutaneously dosage: 5–10 mg/day				
Selegiline	1989	application: orally dosage: 10 mg/day	MAO-B inhibitor	treating motor symptoms	orthostatic hypotension, dizziness, nausea, sleepiness	^292^^,^^570^^,^^571^
Rasagiline	2006	application: orally dosage: 1 mg/day				
Safinamide	2017	application: orally dosage: 50 mg/day				
Zonisamide	2000	application: orally dosage: 25–200 mg/day				
Entacapone	1999	application: orally dosage: 600 mg/day	COMT	treating motor symptoms	orthostatic hypotension, nausea,	^292^^,^^572^
Opicapone	2020	application: orally dosage: 50 mg/day				
Tolcapone	1998	application: orally dosage: 300 mg/day				
Trihexyphenidyl	2003	application: orally dosage: 1–60 mg/day	anticholinergics	treating motor symptoms	cognitive impairment, anxiety, dizziness	^573^
Benztropine	1954	application: orally dosage: 1.5 mg/day				
Amantadine	1973	application: orally dosage: 1–60 mg/day	NMDAR antagonist	ameliorating treatment-induced dyskinesias and extrapyramidal side effects	orthostatic hypotension, hallucinations, gastrointestinal effects	^562^^,^^566^
Deep brain stimulation	2002	-	implantation of electrodes in the subthalamic nucleus or the globus pallidus interna	treating motor symptoms	seizure, infection, headache, confusion, stroke	^564^
Rivastigmine	1997	application: orally or transdermally dosage: 3–12 mg/day (oral) or 4.6–9.5 mg/day (transdermal)	AChE inhibitor	ameliorating PD-related dementia	well-tolerated, gastrointestinal effects	^292^^,^^566^
Citalopram	1998	application: orally dosage: varies	SSRIs, SSNRIs, tricyclic antidepressants	ameliorating PD-related depression	dizziness, headache, akathisia weight gain	^566^^,^^574^
Sertraline	1999					
Paroxetine	1999					
Fluoxetine	1999					
Venlafaxine	1993					
Duloxetine	2004					
Desipramine	1964					
Amitriptyline	1961					
Nortriptyline	1964					
Pimavanserin	2016	application: orally dosage: 10–34 mg/day	5-HT2AR inverse agonist and antagonist	ameliorating PD-related psychosis	confusion, constipation, nausea, weight gain	^12^^,^^289^, ^290^, ^291^
Clozapine	1989	application: orally dosage: varies	multimodal action on multiple neurotransmitter systems	ameliorating PD-related psychosis	extrapyramidal effects, akathisia, dizziness, weight gain	292, 293, 294, 295, 296
Quetiapine	1997					
Olanzapine	1996					
Risperidone	1993					
Aripiprazole	2002					
Ziprasidone	2001					
Melperone	1972					

COMT, catechol O methyltransferase.

In addition to pharmacological intervention, PD symptoms can be treated with deep brain stimulation. Electrodes are surgically implanted in the subthalamic nucleus, the globus pallidus interna, or the zona incerta, regions of the basal ganglia that are involved in the regulation of motor function.^564^

Treatment of nonmotor symptoms is based on targeting neurotransmitters other than dopamine. For example, PD-related dementia can be treated with the AD drug rivastigmine.^565^^,^^566^ Additionally, SSRIs and other antidepressant drugs are commonly used to treat depressive symptoms in PD.^566^ PD-related psychosis is treated with pimavanserin or other antipsychotics.^12^^,^^566^

### D Strategies targeting the serotonergic system

Therapies that selectively or non-selectively target the serotonergic system are currently available for patients with PD. These include MAOIs to treat motor symptoms, and neuroleptics to treat PD-related psychosis (see Table 4). In addition, targeted modulation of the serotonergic system by 5-HTR ligands has been suggested for the treatment of both motor and nonmotor symptoms of patients with PD and for ameliorating the adverse effects of antiparkinsonian medications, such as L-DOPA-induced dyskinesia (Fig. 5). To validate these approaches preclinical models that mimic parkinsonism are used. These are typically based on the administration of neurotoxins, including 6-hydroxydopamine (6-OHDA) and 1-methyl-4-phenyl-1,2,3,6-tetrahydro-pyridine (MPTP),^575^ DR antagonists or inverse agonists, such as haloperidol, monoamine transporter inhibitors, or cholinomimetic agents, such as AChEIs or AChR agonists (Table 5).^576^, ^577^, ^578^, ^579^, ^580^, ^581^, ^582^, ^583^, ^584^, ^585^, ^586^, ^587^, ^588^, ^589^, ^590^, ^591^, ^592^, ^593^, ^594^, ^595^, ^596^, ^597^, ^598^, ^599^, ^600^, ^601^, ^602^, ^603^, ^604^, ^605^, ^606^, ^607^, ^608^, ^609^, ^610^, ^611^, ^612^, ^613^, ^614^, ^615^, ^616^, ^617^, ^618^, ^619^, ^620^, ^621^, ^622^, ^623^, ^624^, ^625^, ^626^, ^627^, ^628^, ^629^, ^630^, ^631^, ^632^, ^633^, ^634^, ^635^, ^636^, ^637^, ^638^, ^639^, ^640^, ^641^, ^642^, ^643^, ^644^, ^645^, ^646^, ^647^, ^648^, ^649^

**Table 5:** Proposed serotonergic interventions in PD

Targeted Pathomechanisms/Symptoms	Action on the Serotonergic System	Drug	References
Dopaminergic deficits	5-HT1AR agonist	8-OH-DPAT	^577^
		R-8-OH-DPAT	577, 578, 579
		Repinotan	^580^
		Flesinoxan	^581^
		S-15535	^581^
		MKC-242	^582^
	5-HT1AR/2AR agonist	5-MeO-DMT	^583^
	5-HT2A/CR agonist	MDMA	583, 584, 585
		DOI	^583^
Glutamatergic overactivation	5-HT2AR antagonist	MDL-100907	^586^
Neuroinflammation/oxidative stress/ROS production	5-HT1AR agonist	8-OH-DPAT	^587^
Mitochondrial dysfunction	MAOI	Rasagiline	588, 589, 590
		Selegiline	
Apoptosis/neuronal loss/cell death	MAOI	Rasagiline	588, 589, 590
		Selegiline	
Dopaminergic neurodegeneration	MAOI	Selegiline	^335^^,^^591^
		TV-3326	
	5-HT1AR agonist	8-OH-DPAT	^587^
		BAY-639044	^592^
		Repinotan	^592^
Motor dysfunction	SSRI	Fluvoxamine	^593^^,^^594^
	MAOI	Rasagiline	^570^^,^^595^
		Selegiline	
		TV-3326	
	5-HT1AR agonist	8-OH-DPAT	596, 597, 598, 599, 600, 601, 602, 603
		R-8-OH-DPAT	^604^^,^^605^
		Buspirone	^600^^,^^603^^,^^606^
		Tandospirone	^601^^,^^607^^,^^608^
		Pardoprunox	^569^^,^^609^, ^610^, ^611^
	5-HT2A/CR agonist	MDMA, R-, S-MDMA	612, 613, 614, 615
	5-HT2AR antagonist	MDL-100907	^586^^,^^616^^,^^617^
	5-HTA/CR antagonist	Ritanserin	^599^^,^^617^, ^618^, ^619^, ^620^, ^621^
		Mirtazapine	^622^^,^^623^
	5-HT2B/CR antagonist	SB-206553	^624^
		Mesulergine	625, 626, 627, 628, 629
	5-HTCR antagonist	SB-228357	^630^
	5-HT3R antagonist	Bemesetron	^631^
		Granisetron	^631^
		Ondansetron	^619^
	5-HT2R antagonists/inverse agonists	Pimavanserin	^632^
		Clozapine	^633^^,^^634^
		Thioridazine	^634^
		Risperidone	^634^
		Mianserin	^635^
	5-HT4R agonist	Cisapride	^636^
	5-HT6R antagonist	SB-258585	619, 620, 621
Hyperalgesia	5-HT3R antagonist	Ondansetron	^637^
Cognitive impairments	MAOI	Rasagiline	^570^^,^^595^
		Selegiline	
		TV-3326	
Psychosis	5-HT2R antagonists/inverse agonists	Pimavanserin	^12^^,^^289^
		Clozapine	292, 293, 294, 295, 296
		Quetiapine	
		Olanzapine	
		Risperidone	
		Aripiprazole	
		Ziprasidone	
		Melperone	
	5-HT3R antagonist	Ondansetron	^638^^,^^639^
Depression	SSRI	Paroxetine	^296^^,^^574^
		Venlafaxine	^296^
	MAOI	Rasagiline	^570^^,^^595^
		Selegiline	
		TV-3326	
Anxiety	MAOI	Rasagiline	^570^^,^^595^
		Selegiline	
		TV-3326	
Apathy	MAOI	Rasagiline	^570^^,^^595^
		Selegiline	
		TV-3326	
Gastrointestinal dysfunction	5-HT4R agonist	Cisapride	640, 641, 642
		Mosapride	^643^^,^^644^
		Tegaserod	^645^^,^^646^
L-DOPA-induced dyskinesia	5-HT1AR agonist	NLX-112	NCT05148884
		Buspirone	^647^
		Mirtazapine	^648^
		Sarizotan	^649^

5-MeO-DMT, 5-methoxy-N, N-dimethyltryptamine.

#### 1 Modulation of 5-HT levels

As mentioned in section *IV B*, 5-HT levels are decreased in patients with PD,^499^^,^^500^^,^^502^^,^^503^ which correlate with the severity of both motor symptoms, eg, tremor, rigidity, and akinesia, and nonmotor symptoms, eg, depression and pain.^504^^,^^650^^,^^651^ Therefore, drugs that increase cellular 5-HT levels, such as SSRIs and MAOIs, might be beneficial in the treatment of PD. However, the use of SSRIs in PD is highly debated. Although the SSRI fluvoxamine in combination with the dopamine reuptake inhibitor GBR-12909 or the unspecific reuptake inhibitor mazindol had beneficial effects on motor function in WT and 6-OHDH-lesioned rats,^593^^,^^594^ several studies showed that SSRIs, eg, sertraline, fluoxetine, and paroxetine, potentially worsened motor symptoms in PD.^621^^,^^652^, ^653^, ^654^, ^655^, ^656^, ^657^, ^658^, ^659^

As depression is a common symptom of PD, SSRIs and other antidepressants are frequently taken by patients with PD. A retrospective study involving 221 patients with PD found no increased risk of worsening motor symptoms compared to nonserotonergic antidepressants.^660^ In addition, the SSRI paroxetine and the serotonin-norepinephrine reuptake inhibitor venlafaxine have been reported to be clinically effective against depressive symptoms in patients with PD without worsening motor function.^296^^,^^574^ In contrast, other studies have questioned the efficacy of various SSRIs, such as citalopram, sertraline, paroxetine, and fluoxetine, in the treatment of depression in PD due to insufficient evidence.^661^^,^^662^

MAOIs are useful in the treatment of nonmotor symptoms, such as cognition, depression, and anxiety (see section *IV C* for details). In addition, the selective MAO-B inhibitors rasagiline and selegiline also protect against neuronal loss by stabilizing the mitochondrial membrane potential and preventing caspase-3-mediated DNA fragmentation.^588^, ^589^, ^590^ Consistently, selegiline and TV-3326, an MAOI and ChE inhibitor, prevented nigrostriatal and substantia nigral degeneration in MPTP-treated PD animal models.^335^^,^^591^ In clinical studies, rasagiline, selegiline, and TV-3326 had beneficial effects on motor fluctuations, cognition, depression, anxiety, apathy, and quality of life in adjuvant treatment with L-DOPA, highlighting its therapeutic potential in PD.^570^^,^^595^

#### 2 Targeting the 5-HT1R

Several studies suggest that targeting the 5-HT1AR may modulate dopamine release. Activation of 5-HT1AR by highly selective agonists, such as 8-OH-DPAT, R-8-OH-DPAT, repinotan, flesinoxan, S-15535, and MKC-242, increased neuronal dopaminergic activity and dopamine release by blocking GABAergic neurons in the prefrontal cortex, nucleus accumbens, and ventral tegmental area of rodents.^577^, ^578^, ^579^, ^580^, ^581^, ^582^ Furthermore, the 5-HT1AR agonists 8-OH-DPAT, BAY-639044, and repinotan protected primary cultured astrocytes from oxidative stress and prevented neurodegeneration in the substantia nigra and striatum of 6-OHDA-lesioned and MPTP-treated rodent PD models,^587^^,^^592^ highlighting the antiparkinsonian properties of the 5-HT1AR.

Indeed, there is convincing evidence that activation of 5-HT1AR improves motor function in various animal models of PD. Selective and partial agonists of the 5-HT1AR, including 8-OH-DPAT and the anxiolytics buspirone and tandospirone, have been shown to alleviate catalepsy and bradykinesia induced by either the D2R/D3R inverse agonist haloperidol or by 6-OHDA-lesioning in rodents.^596^^,^^598^, ^599^, ^600^, ^601^, ^602^, ^603^^,^^606^^,^^608^^,^^620^^,^^663^ In addition, activation of the 5-HT1AR by R-8-OH-DPAT, tandospirone (a selective 5-HT1AR agonist) or pardoprunox (a combined 5-HT1AR agonist and partial D2R/D3R agonist) abolished locomotor deficits induced by monoamine transporter inhibitors in rodents.^604^^,^^611^^,^^663^ Consistently, pardoprunox dose-dependently enhanced locomotor activity in a primate animal model of PD.^610^^,^^611^ 5-HT1AR activation by various agonists, eg, 8-OH-DPAT, R-8-OH-DPAT, tandospirone, and pardoprunox, also improved rotational behavior and parkinsonian tremor in unilateral 6-OHDA-lesioned rodents, probably mediated by enhanced dopamine release or postsynaptic DR stimulation.^597^^,^^605^^,^^611^^,^^663^^,^^664^ Although some of the aforementioned compounds do not exclusively target 5-HT1AR, their importance for these antiparkinsonian effects has been confirmed by the additional use of 5-HT1AR antagonists. Mechanistically, these effects are mainly attributed to the activation of postsynaptic 5-HT1ARs, particularly in the cortex and striatum.^582^^,^^601^^,^^602^^,^^604^^,^^605^^,^^608^^,^^658^^,^^665^

5-HT1AR activation has also shown promising results in clinical trials. For example, pardoprunox significantly improved motor symptoms in patients with PD compared with placebo.^569^^,^^609^ However, it is not clear whether the effects were attributable to the action on the dopaminergic, the serotonergic, or both systems. In addition, the 5-HT1AR agonists buspirone, mirtazapine, and sarizotan effectively reduce the L-DOPA-induced dyskinesia in patients with PD.^647^, ^648^, ^649^ More recently, a phase 2a clinical trial evaluated the safety, tolerability, and preliminary efficacy of the highly selective 5-HT1AR agonist Befiradol (NLX-112) in PD patients with L-DOPA-induced dyskinesia, which is effective in MPTP-treated nonhuman primates without exacerbating parkinsonian symptoms.^666^ This randomized, double-blind, placebo-controlled study confirmed that NLX-112 was well-tolerated and significantly reduced dyskinesia severity compared to placebo (NCT05148884). Further clinical trials are planned to assess its long-term efficacy and safety.

#### 3 Targeting the 5-HT2R

The 5-HT2R has emerged as a promising target in PD to treat both motor and nonmotor symptoms. Activation of the 5-HT2R has been suggested to counteract dopaminergic degeneration in PD by stimulating dopamine release. MDMA, which stimulates the 5-HT2R, increases striatal extracellular dopamine concentration in vivo. This was further enhanced by specific 5-HT2R agonists, such as 2,5-dimethoxy-4-iodophenyl (DOI) and 5-methoxy-N, N-dimethyltryptamine, and alleviated by the application of 5-HT2R antagonists, such as ketanserin and ritanserin.^583^, ^584^, ^585^ Consistent with this, several studies provide evidence that activation of the 5-HT2R by MDMA may be effective in the treatment of motor symptoms, including catalepsy, locomotor activity, and sensorimotor function in different PD animal models.^612^, ^613^, ^614^, ^615^ Interestingly, while both 5-HT2R subtypes, the 5-HT2AR, and 5-HT2CR, jointly contribute to the anticataleptic action, rotational behavior seems to be mediated mainly by the 5-HT2CR.^612^

In contrast, several studies demonstrated that blocking the 5-HT2R had beneficial effects on motor function. For example, the 5-HT2R antagonists SB-228357 and ritanserin rescued haloperidol-induced catalepsy and bradykinesia in rodents.^630^^,^^599^^,^^619^, ^620^, ^621^ Mechanistically, specific blockade of the 5-HT2CR subtype seems to mediate the anticataleptic effect, probably by blocking its inhibitory effects on dopamine release.^560^^,^^630^ In addition, the 5-HTCR antagonist SB-206553 has been shown to induce rotation in the PD animal model.^624^ Furthermore, 5-HT2R antagonists, including MDL-100907, ketanserin, and ritanserin, abolished hyperactivity in 6-OHDA-lesioned rats and improved sensorimotor performance in MPTP-treated mice.^586^^,^^616^^,^^617^ In contrast to cataleptic behavior, locomotor behavior and sensorimotor function might be mainly regulated by 5-HT2AR rather than 5-HT2CR by restoring the overactivated glutamatergic system.^586^^,^^617^ Furthermore, blocking the 5-HT2R by either antagonists or inverse agonists, such as pimavanserin, clozapine, thioridazine, risperidone, and mianserin, has been shown to have antitremor effects in different animal models of PD.^632^, ^633^, ^634^, ^635^

5-HT2R blockade also shows some promising effects in patients with PD. Mesulergine is a multimodal drug acting as a 5-HT2R antagonist and has been shown to have antiparkinsonian effects, including improved tremor, rigidity, bradykinesia, akinesia, and gait.^625^, ^626^, ^627^, ^628^, ^629^^,^^667^, ^668^, ^669^ Although the treatment did not worsen dyskinesia in patients,^627^^,^^668^ mesulergine caused histological changes in rats, leading to discontinuation of clinical studies.^627^ Another nonselective 5-HT2A/CR antagonist, mirtazapine, also alleviated tremor symptoms in patients with PD.^622^^,^^623^ Additionally, although the nonselective 5-HT2A/CR antagonist ritanserin improved akinesia and gait in clinical studies, tremor was not significantly ameliorated compared with placebo control subjects.^618^ Besides motor symptoms, inhibition of the 5-HT2R can ameliorate PD psychosis, including visual hallucinations and delusions. Several atypical antipsychotics that block the 5-HT2R are already used in the treatment of PD psychosis (see section *IV C*). For example, the selective 5-HT2AR inverse agonist and antagonist pimavanserin had beneficial effects on psychosis without worsening motor functions in PD patients with psychosis.^12^^,^^289^ Based on these initial observations, an observational clinical trial was recently started to validate the efficacy of pimavanserin in the treatment of impulse control disorders in PD (RETRO-PIMPARK; NCT06754553).

The fact that both agonists and antagonists of the 5-HT2R can have beneficial effects on PD motor symptoms may be explained by the receptor's narrow optimal activation range. Even minor deviations, either above or below the optimal level, could contribute to motor dysfunction. Depending on factors such as the study design, the specific PD model used, disease stage at treatment initiation, and treatment duration may influence receptor activity, potentially shifting it outside the functional range. In cases where receptor activity is excessively high or low, the administration of either agonists or antagonists may help restore it to an optimal level, thereby alleviating motor symptoms.

#### 4 Targeting the 5-HT3R

Although the role of 5-HT3R in PD has not been well studied yet, there are a few studies indicating the therapeutic potential of 5-HT3R antagonists in the treatment of motor and nonmotor symptoms. Treatment with the 5-HT3R antagonists and antiemetics bemesetron, granisetron, or ondansetron improved catalepsy and bradykinesia in rodents,^619^^,^^631^ while ondansetron alleviated PD-related hyperalgesia in 6-OHDA-lesioned rats.^637^ Furthermore, a clinical study demonstrated that ondansetron improved PD-related psychosis without affecting motor symptoms and cognitive function in patients with PD.^638^^,^^639^ A follow-up phase 2 study, completed at the end of 2024, further confirmed the effects of ondansetron treatment on hallucinations (NCT04167813).

#### 5 Targeting the 5-HT4R

In PD, the focus of 5-HT4R targeting has been on the nonmotor symptom of gastrointestinal dysfunction. Stimulation of the 5-HT4R with the agonists cisapride, mosapride, or tegaserod improved PD-related gastrointestinal dysfunction, including constipation and reduced gastric motility in patients with PD.^640^, ^641^, ^642^, ^643^, ^644^, ^645^, ^646^ However, whether 5-HT4R activation might improve motor function remains elusive. Sempere et al^670^ observed a deterioration of tremors upon cisapride treatment. Another study reported improved visuomotor coordination and gait function.^636^ However, since the patients in this study were cotreated with L-DOPA, the cisapride-mediated increase in gastric motility is likely to enhance L-DOPA absorption, thereby increasing the effect of L-DOPA treatment.^636^

More recently, an exploratory study was initiated to assess 5-HT4R expression at a moderate stage of PD using PET imaging with the selective radioligand [^11^C]SB207145. In parallel, the study monitors the impact of the BDNF on the 5-HT4R expression (NCT05916625).

#### 6 Targeting the 5-HT6R

Some studies suggest that blocking the 5-HT6R may have antiparkinsonian effects. In particular, the 5-HT6R antagonist SB-258585 alleviated catalepsy and bradykinesia in rodents.^619^, ^620^, ^621^

#### 7 Targeting the 5-HT7R

Blocking the 5-HT7R has also been proposed to be beneficial in PD. Pirepemat (IRL752) is a novel drug in development designed to reduce falls in patients with PD by enhancing neural signaling in the prefrontal cortex through antagonism of the 5-HT7R and *ɑ*-2 receptors. This mechanism aims to increase dopamine and noradrenaline levels, thereby addressing the balance impairments associated with cognitive decline in PD.^671^ A phase 2b clinical trial, REACT-PD (NCT05258071), is currently evaluating pirepemat’s efficacy, safety, and tolerability in reducing fall frequency in patients with PD. The study is ongoing at multiple European sites, and top-line results are expected in the first quarter of 2025. In addition, a phase 4 clinical trial was initiated in early 2025 to evaluate the efficacy of 5-HT7R antagonist vortioxetine in reducing freezing of gait symptoms in patients with PD with depressive symptoms unresponsive to dopaminergic treatment (NCT06805266).

In summary, numerous preclinical and clinical studies provide compelling evidence that specific targeting of the serotonergic system might be beneficial for improving motor function and ameliorating neuropsychiatric symptoms in PD. However, research in this area should be accelerated to provide PD patients with new therapeutic approaches.

## V Amyotrophic lateral sclerosis

### A Disease-specific pathological phenotypes

ALS is a rare disease with an estimated global prevalence of 4.4 per 100,000 persons, which is higher among men than women (Table 1).^91^ This fatal neurodegenerative disease affects upper and lower motor neurons in the motor cortex, brainstem, and spinal cord.^98^ Progressive loss of these neurons causes deterioration of muscle control, spasticity, muscle atrophy, and paralysis. Weight loss, fatigue, emotional lability, and cognitive dysfunction are other common symptoms. Survival varies from 2 to 10 years, with the majority of patients dying from respiratory failure within 3 years of symptom onset.^96^

Besides genetic factors (Table 1), diverse molecular mechanisms contribute to motor neuron degeneration in ALS. One of the most important pathological mechanisms is glutamate-induced excitotoxicity, which is driven by excessive activation of glutamate receptors and causes acute neuronal swelling, increased Ca^2+^ influx, and the production of free radicals, resulting in neuronal death.^672^, ^673^, ^674^, ^675^, ^676^ Importantly, human motor neurons seem to be more susceptible to glutamate toxicity caused by lower levels of the AMPAR GluR_2_ subunit and the expression of atypical glutamate receptors associated with a reduced ability in Ca^2+^ buffering.^677^, ^678^, ^679^, ^680^

Another central disease mechanism in ALS is the aggregation and subcellular mislocalization of the nuclear DNA/RNA-binding proteins TDP-43 and FUS. The majority of patients with ALS exhibit cytoplasmic inclusions of hyperphosphorylated and ubiquitinated TDP-43, often accompanied by its nuclear clearance,^77^^,^^99^ while deposits of FUS have been observed in a more restricted subtype of ALS.^101^^,^^681^ Furthermore, aggregates of the SOD1 enzyme can be found in patients with ALS with *SOD1* mutations,^102^ and the accumulation of insoluble polypeptides is specific to carriers of an intronic hexanucleotide repeat expansion in the *C9orf72* gene.^100^ To date, it is not fully understood how the aggregation of these proteins drives motor neuron degeneration, but TDP-43, FUS, and SOD1 dysfunction, and *C9orf72* mutations have been associated with abnormal RNA metabolism,^682^^,^^683^ impaired DNA repair,^684^, ^685^, ^686^, ^687^ dysregulated nucleocytoplasmic and axonal transport,^688^, ^689^, ^690^, ^691^ and compromised mitochondrial function.^692^, ^693^, ^694^, ^695^ Notably, NFTs and Lewy bodies have also been observed in brain samples of patients with ALS.^696^^,^^697^

Further processes have been described in ALS pathology that either contribute to or are a consequence of pathological protein aggregation, eg, oxidative stress,^698^, ^699^, ^700^ impaired autophagy,^701^ and stress granule formation.^702^ In addition, non-neuronal cells, including astrocytes, microglia, and oligodendrocytes, are critically involved in disease onset and progression.^703^, ^704^, ^705^, ^706^, ^707^, ^708^

### B Serotonergic imbalances

**Fig. 6:**
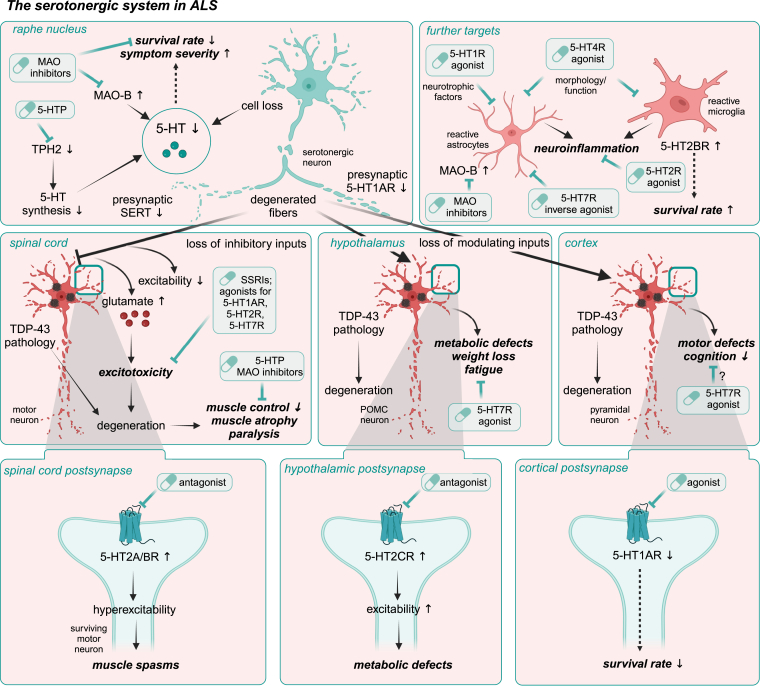
Serotonergic deficiencies in ALS and its therapeutic targeting. Overview of serotonergic changes in the brain and spinal cord during the progression of ALS. The loss of serotonin-producing neurons in the raphe nucleus, as well as degenerated ascending and descending serotonergic projections, is evident even in the presymptomatic stages of the disease (upper left panel). The underlying mechanism responsible for the degeneration of these neurons remains to be elucidated, as the presence of TDP-43 pathology is not detected in serotonergic neurons. Impaired synthesis by raphe neurons and increased degradation by reactive astrocytes further contribute to reduced serotonin levels in patients with ALS, which correlate with symptom severity and lower survival rates. The loss of inhibitory serotonergic input in the spinal cord directly affects the excitability and glutamate neurotransmission of motor neurons and may cause—in combination with TDP-43 pathology—motor neuron degeneration and the manifestation of motor impairment (middle left panel). The loss of serotonergic projections is compensated by increased 5-HT2R expression in the spinal cord and hypothalamus, resulting in typical ALS symptoms, such as muscle spasms and weight loss (lower left and middle panels). Impaired serotonergic neurotransmission in the cortex may contribute to motor deficits and cognitive decline (middle right panel). In addition to neuronal degeneration, reactive astrocytes and microglia drive neuroinflammation in the affected brain regions, further exacerbating disease progression (upper right panel). The potential direct consequences of serotonergic alterations are indicated by arrows with solid lines, and correlations by arrows with dashed lines. Proposed targeted interventions for serotonergic imbalances are highlighted by green boxes with a pill symbol.

Changes in the serotonergic system are also prominent in ALS (Fig. 6). Decreased levels of 5-HT and its precursor TRP are found in the CSF, serum, and plasma, as well as platelets of patients with ALS.^709^, ^710^, ^711^ However, studies using postmortem human tissue samples have produced contradictory results: while some identified lower levels of 5-HT and its metabolite 5-HIAA in the spinal cord of patients with ALS,^712^ others reported normal levels of 5-HT but changes in 5-HIAA.^713^^,^^714^ This inconsistency might result from the low stability of these molecules and different postmortem delay times. Importantly, abnormal lower 5-HT levels correlate with symptom severity and reduced survival in patients with ALS.^710^^,^^713^ Studies in different animal models of ALS provide further evidence of reduced 5-HT levels in the early and late stages of the disease.^13^^,^^715^^,^^716^

5-HT depletion in ALS has been suggested because of the loss of serotonergic neurons. Indeed, degeneration of central serotonergic neurons, characterized by loss of neuritic processes and cell bodies in the brainstem and reduced numbers of serotonergic projections to the spinal cord and the hippocampus, has been observed in patients with ALS.^13^ Noteworthy, serotonergic neurons do not exhibit typical TDP-43 pathology.^13^ The reason for this is not yet clear, but there are several possible explanations: serotonergic neurons (1) generally produce a lower amount of aggregates, (2) have a higher capacity to clear aggregates, or (3) are more sensitive to even low amounts of aggregates.^717^ Changes with the 5-HT system have also been observed in different animal models of ALS. Most studies are consistent with the findings in patients, showing a decreased number of serotonergic fibers in different brain regions, including the brainstem and spinal cord.^13^^,^^716^^,^^718^^,^^719^ These deficiencies occur already before the onset of motor symptoms and persist during disease progression,^13^^,^^716^ highlighting an involvement of the serotonergic system early in the pathogenesis of ALS. Reduced levels of TPH2 and SERT have also been found in affected brain regions of ALS mice,^13^^,^^715^^,^^719^ which could either directly contribute to cellular 5-HT depletion or could result from the degeneration of central serotonergic neurons. In addition, higher expression of MAO-B on reactive astrocytes in the spinal cord and affected brain regions promotes fast degradation of 5-HT.^720^^,^^721^

5-HT is a potent neuromodulator that can increase the excitability of motor neurons.^722^ Thus, the depletion of 5-HT has a direct effect on the function of motor neurons and may consequently contribute to their degeneration. Interestingly, motor neurons with dense serotonergic innervation appear to be more susceptible to degeneration in ALS than motor neurons with sparse 5-HT inputs.^723^ Degeneration of serotonergic projections in ALS has also been associated with an increased glutamate-induced excitotoxicity. 5-HT inhibits glutamate transmission in different regions of the central nervous system, including those involved in the control of motor functions, by activating different 5-HTRs, such as 5-HT1AR, 5-HT1BR, 5-HT2R, and 5-HT6R.^724^ A loss of serotonergic projections might be compensated by increased glutamatergic input leading to overexposure to neurotoxic amounts of glutamate.^713^ Melatonin, a serotonin metabolite, has also been shown to inhibit glutamate release and protect from neurotoxicity.^725^^,^^726^ Low melatonin levels are caused by reduced 5-HT, and therefore, could further contribute to glutamate-induced excitotoxicity and motor neuron loss in ALS.

Serotonergic neurotransmission in ALS is compromised not only by lower 5-HT concentrations but also at the receptor level. Although not yet systematically studied, alterations in the expression of specific 5-HTR subtypes have been frequently observed in patients with ALS, although findings vary depending on the CNS regions examined and the methods used. Autoradiography with [^3^H]-8-OH-DPAT, a 5-HT1AR full agonist, in postmortem tissue of patients with ALS revealed increased 5-HT1AR levels in the cervical, thoracic, and lumbar spinal cord.^727^ In contrast, using the same method, Forrest et al^714^ reported lower levels of 5-HT1AR in the cervical spinal cord, while no changes were detected in the lumbar spinal cord as well as the motor and premotor cortex of patients with motor neuron disease. Reduced levels of 5-HT1AR in the raphe nuclei and frontotemporal regions of the cortex have been identified in patients with sporadic ALS using [^11^C]-WAY100635 PET. Importantly, the reduction was more pronounced in patients with a bulbar onset, which typically correlates with poorer survival, than in those with a limb onset.^728^ Consistent with this, cortical 5-HT1AR levels were decreased in patients with the SOD1^D90A^ mutation. Compared with patients with sporadic ALS, the reduction was less pronounced and correlates with an above-average survival of 14 years in these patients.^729^ While the decreased density of 5-HT1AR in the raphe nuclei may result from degeneration of central serotonergic neurons, the cellular processes driving the loss of postsynaptic 5-HT1AR are still unclear.

Besides 5-HT1AR, altered expression of 5-HT2R has also been reported in patients and ALS animal models. Autoradiography with [^3^H]-ketanserin, a highly selective 5-HT2AR antagonist, revealed lower receptor density in the premotor cortex of postmortem tissue from patients with motor neuron disease, while 5-HT2AR levels in the spinal cord and the motor cortex were unchanged compared to controls.^714^ In line with this, immunohistochemistry shows no significant differences in the density of 5-HT2AR in the cervical spinal cord of SOD1^G93A^ rats at a late stage of the disease.^730^ However, significantly lower levels of 5-HT2AR were found at a presymptomatic stage, suggesting an increase in the 5-HT2AR expression during disease progression. Similar findings from various ALS animal models have been reported for the other 2 5-HT2R subtypes. While 5-HT2BR is strongly upregulated on surviving motor neurons in the spinal cord and brainstem, as well as on activated microglia,^13^^,^^730^, ^731^, ^732^ elevated levels of 5-HT2CR were found in hypothalamic proopiomelanocortin neurons, which are involved in the regulation of appetite and energy homeostasis.^718^^,^^733^ Upregulation of these receptors might compensate for the loss of serotonergic neurons to maintain neuronal excitability and plasticity, facilitate microglial mobility, and regulate metabolic defects. The identification of a rare allelic variant of the SNP rs10199752 in the human *HTR2B* gene further supports that 5-HT2BR upregulation might play a protective role in ALS. Compared with the carriers of the common A allele, patients with ALS with one C allele have higher 5-HT2BR mRNA levels in the spinal cord and less degeneration of mononuclear phagocytes, which correlates with longer survival.^731^ However, increased expression of these receptors has also been associated with neuronal hyperexcitability and muscle spasms.^13^^,^^732^^,^^734^

Whether the serotonergic dysfunctions observed in patients and transgenic animal models of the disease are primary drivers of ALS pathogenesis or rather a secondary event of motor neuron degeneration remains elusive. However, several findings highlight a central role of the serotonergic system in ALS onset and progression (Fig. 6). First, serotonergic alterations occur already in a nonsymptomatic phase long before the onset of motor symptoms.^13^ Some studies imply that serotonergic dysfunction may even begin at embryonic stages, similar to abnormal motor neuron activity.^716^^,^^735^ Second, symptom severity and survival of patients correlate with the degree of serotonergic dysfunction, suggesting that longer preservation of serotonergic projections slows down disease progression.^728^^,^^729^ Third, novel genetic variants of genes involved in serotonin synthesis and metabolism (*TPH1, TPH2*, and *MAOA*) have been recently identified in patients with sporadic ALS, which might contribute to the risk of developing the disease.^736^

### C Current treatment strategies

To date, 4 disease-modifying therapies have been approved for ALS, targeting different pathophysiologic disease mechanisms (Table 6).^737^, ^738^, ^739^, ^740^, ^741^, ^742^, ^743^, ^744^, ^745^, ^746^, ^747^, ^748^, ^749^, ^750^, ^751^, ^752^, ^753^, ^754^ Riluzole, which counteracts glutamate excitotoxicity, is used by the majority of patients with ALS. Clinically, riluzole has been shown to increase survival, particularly in patients with bulbar onset and late-stage disease, while only marginally improving motor symptoms.^737^, ^738^, ^739^, ^740^ Another ALS drug is Edaravone, a low molecular weight antioxidant.^741^ Because of its controversial clinical efficacy, it is only available in Japan and the U.S.^742^, ^743^, ^744^, ^745^, ^746^, ^747^, ^748^ AMX0035, also known as PB-TURSO, which is a mixture of sodium phenylbutyrate and taurursodiol, induces antiapoptotic signaling and protects cells from mitochondrial damage.^749^^,^^750^ AMX0035 has been shown to slow down disease progression and functional decline and to prolong survival in patients with ALS.^751^^,^^752^ More recently, the US Food and Drug Administration approved tofersen for the treatment of patients with ALS carrying *SOD1* mutations. This antisense oligonucleotide targets *SOD1* mRNA to reduce SOD1 protein synthesis. First clinical trials confirmed reduced SOD1 concentrations and decreased plasma concentration of neurofilament light chain, a marker for neurodegeneration in the CSF of the tofersen-treated group.^753^^,^^754^

**Table 6:** Current treatments for ALS

Drug	Approval	Prescription	Mode of Action	Clinical Efficacy	Adverse Effects	References
Riluzole	1995	application: orally as a tablet, suspension, and a sublingual disintegrating film dosage: 100 mg/ day	probably counteracting glutamate excitotoxicity by blocking voltage-gated sodium channels	increasing survival, slowing down disease progression and functional decline	well-tolerated	737, 738, 739, 740^,^^755^^,^^756^
Edaravone	2017 not in Europe	application: orally or intravenously dosage: 105 mg/month	unknown; presumably protecting from oxidative stress by targeting free radicals	ranging from no effects to improving disease progression, functional scores, and survival	bruising, gait disturbances, headaches, eczema	741, 742, 743, 744, 745, 746, 747
AMX0035 (PB-TURSO)	2022	application: orally dosage 3 g sodium phenylbutyrate and 1 g taurursodiol/day	antiapoptotic signaling, protecting from mitochondrial damage	increasing survival, slowing down disease progression and functional decline	well-tolerated, gastrointestinal effects	749, 750, 751, 752
Tofersen	2023	application: intrathecally dosage: 3 initial 100 mg doses followed by a maintenance dose	antisense oligonucleotide targeting *SOD1* mRNA	reducing SOD1 levels and neuroinflammation	pain, fatigue, increased CSF white blood cells	^753^^,^^754^

In addition to the available disease-modifying drugs, symptomatic therapies are an important part of the treatment of ALS. Different pharmacological and nonpharmacological approaches are currently used to manage the consequences of respiratory failure and malnutrition, as well as to improve pain, muscle cramps, spasticity, mood disturbances, and fatigue.^98^

### D Strategies targeting the serotonergic system

Despite the remarkable serotonergic dysfunctions observed in ALS, no treatments targeting the 5-HT system are currently available for patients with ALS. However, accumulating studies provide evidence that targeting the serotonergic system, either by increasing 5-HT levels or by modulating 5-HTR activities, may be a promising strategy to combat both disease-related pathophysiological mechanisms and their secondary consequences in patients with ALS (Fig. 6; Table 7).^757^, ^758^, ^759^, ^760^, ^761^, ^762^, ^763^, ^764^, ^765^, ^766^, ^767^, ^768^, ^769^, ^770^, ^771^, ^772^, ^773^, ^774^, ^775^, ^776^, ^777^, ^778^, ^779^, ^780^, ^781^, ^782^, ^783^, ^784^, ^785^

**Table 7:** Proposed serotonergic interventions in ALS

Targeted Pathomechanisms/Symptoms	Action on the Serotonergic System	Drug	References
Disease progression/survival	5-HT levels	5-HTP	^757^
	SSRI	Fluoxetine	^758^
	MAOI	Rasagiline	759, 760, 761, 762
		Deprenyl (selegiline)	763, 764, 765, 766
	5-HT1AR agonist	Xaliproden (SR57746A)	767, 768, 769, 770
Glutamate excitotoxicity	SSRI	Paroxetine	^771^
	5-HT1AR agonist	8-OH-DPAT	^772^^,^^773^
	5-HT1BR agonist	CP93129	^773^
	5-HT2R agonist	DOI	^772^
	5-HT7R agonist	8-OH-DPAT	774, 775, 776
Spasticity	5HT2B/CR inverse agonist	SB-206553	^13^^,^^734^^,^^777^
		Cyproheptadine	
Phrenic motor neuron plasticity (breathing dysfunctions)	5-HT2R agonist	DOI	^730^^,^^778^
	5-HT7R agonist	8-OH-DPAT	^779^
Metabolic dysfunctions	SSRI	Fluoxetine	^718^
	5-HT2R antagonist	Olanzapine	^718^^,^^780^
Swallowing dysfunction	5-HT levels	SSRIs, 5-HT	^781^
Neuroinflammation	5-HT2BR	agonist/antagonist?	^731^
	5-HT4R agonist	5-HT, BIMU8	^782^^,^^783^
Depression	SSRI	eg, fluoxetine, escitalopram	^784^^,^^785^

#### 1 Modulation of 5-HT levels

As highlighted in section *V B*, serotonergic denervation is associated with the loss of inhibitory inputs for glutamate release, which contributes to glutamate-induced motor neuron degeneration. Consequently, restoring 5-HT to normal levels may protect ALS motor neurons from glutamate excitotoxicity. In line with this, systemic administration of the 5-HT precursor 5-HTP delays ALS onset, improves survival and increases motor functions in the transgenic SOD1^G93A^ mice.^757^ Furthermore, chronic treatment with the SSRI paroxetine in rats increased the surface expression of the AMPAR GluR2 subunit at the synapse, which may protect motor neurons from glutamate-induced neurotoxicity.^771^ However, long-term treatment with the SSRI fluoxetine in adult SOD1^G93A^ mice did not significantly affect disease progression. The rate of motor impairment was even increased when treatment was initiated at a neonatal stage, highlighting the importance of strictly controlled extracellular 5-HT levels.^758^

Another approach to enhance 5-HT levels is to prevent its degradation by targeting the MAO. MAO-B inhibitors, such as deprenyl/selegiline and rasagiline, are neuroprotective and improve motor function as well as survival in different ALS mouse models.^762^^,^^765^^,^^766^ Despite their promising effects in mice, clinical studies in patients have not been encouraging. Most studies showed no significant effects of MAO-B inhibitors on disease progression and functional measures.^759^^,^^761^^,^^763^^,^^764^^,^^786^ However, rasagiline may be beneficial in a specific subset of patients with ALS with moderate to fast disease progression, in which prolonged survival and reduced functional decline were observed upon treatment.^760^ Rasagiline also changed the biomarker profile in patients with ALS, indicating improvement of mitochondrial function and reduction of oxidative stress and apoptosis.^759^ These effects may be explained by its inhibiting effect on MAO-B expressed on reactive astrocytes and warrant further investigations.^763^

Besides targeting disease-related mechanisms, compensating for 5-HT deficiency may also be a promising strategy for symptomatic therapies in patients with ALS. Brain-derived 5-HT is critically involved in swallowing function, as shown by a study in *TPH2* knockout mice, which have significantly slower licking and swallowing rates.^781^ A supplemental 5-HT therapy might therefore improve swallowing dysfunctions in patients with ALS. Although depression is less common in ALS compared with other neurodegenerative diseases, treatment with SSRIs is also a useful therapy for patients with ALS who have depressive symptoms.^784^

Notably, besides approaches directly targeting the central 5-HT system, new strategies have been proposed to modulate 5-HT levels in the brain by manipulating the gut microbiota.^709^

#### 2 Targeting the 5-HT1R

Activation of the 5-HT1R by specific agonists has been proposed to partly compensate for the loss of 5-HT1R in ALS and to counteract glutamate excitotoxicity. This is mediated in 2 different ways: while activation of 5-HT1AR attenuates postsynaptic glutamate receptor signaling, 5-H1BR inhibits glutamate release at the presynapse.^773^ In line with this, the application of the 5-HT1AR-specific agonist 8-OH-DPAT protected cortical neuronal cultures from glutamate-induced neurotoxicity.^772^ Another selective 5-HT1AR agonist, xaliproden or SR57746A, has been shown to promote neurite outgrowth and survival of motor neurons and stimulate the synthesis of neurotrophic factors by astrocytes.^768^^,^^769^ It also significantly improved survival and ameliorated motor symptoms in a mouse model of motor neuron disorder, highlighting its potential for ALS treatment.^767^ However, the results of clinical phase 3 trials with xaliproden in patients with ALS were not convincing.^770^ Although slight improvements in functional measures and survival were observed, the effects were not significant enough to justify the use of xaliproden, either as a monotherapy or in combination with riluzole, in clinical practice.

#### 3 Targeting the 5-HT2R

The 5-HT2R is the best-studied serotonergic target in ALS. Both activation and blockade of its activity have been proposed for disease-modifying as well as symptomatic therapy.

Activation of 5-HT2R by the selective 5-HT2AR agonist DOI has been shown to protect cultured cortical neurons from glutamate-induced neurotoxicity.^772^ Moreover, an ongoing combined phase 2 or 3 multiarm clinical trial with an adaptive design is investigating whether treatment with the 5-HT2AR antagonist trazodone can slow the progression of motor neuron disease and improve patient survival (NCT04302870). As the 5-HT2BR is critically involved in microglial function by regulating its motility, phagocytic activity, and release of endosomes,^782^^,^^787^ and deletion of the *HTR2* gene exacerbated disease progression in a mouse model of ALS,^731^ activation of the 5-HT2BR has also been suggested as a potential strategy to modulate neuroinflammation in ALS. In addition, 5-HT2R activity has been implicated in the maintenance of respiratory function during disease progression by promoting phrenic nerve plasticity.^730^^,^^778^ However, the evaluation of the specific 5-HT2BR agonist BW723C86 in a fast-progressing transgenic mouse model of ALS did not reflect the promising results of these studies. Although BW723C86 showed favorable pharmacokinetic and pharmacological profiles and confirmed a modulatory role of the receptor in microglial gene expression, no effects on survival, motor function, and motor neuron degeneration were observed upon treatment.^788^

As mentioned in section *V B*, the 5-HT2R subtypes 5-HT2BR and 5-HT2CR are upregulated on surviving motor neurons, which has been associated with neuronal hyperexcitability and muscle spasms.^13^^,^^732^^,^^734^ Thus, blocking the 5-HT2R activity has been suggested to target spasticity in patients with ALS. The application of either broad-spectrum or isoform-specific 5-HT2R inverse agonists, such as cyproheptadine and SB206553, has been shown to reduce muscle spasms in ALS mouse models as well as in paraplegic patients with spinal cord injuries.^13^^,^^734^^,^^777^ In addition, blocking 5-HT2R activity was proposed to compensate for the increased 5-HT2CR expression on hypothalamic proopiomelanocortin neurons, thereby ameliorating metabolic defects observed in patients with ALS.^718^ Beneficial effects in mice have been reported with the antipsychotic olanzapine, which also acts as a nonspecific 5-HT2CR antagonist, leading to increased food intake and weight gain.^780^ Importantly, a high-energy diet significantly increased survival and motor neuron function in transgenic ALS mice, indicating that compensation of metabolic defects by 5-HT2CR antagonists might be more than just a symptomatic therapy.^789^ A clinical study investigating the effects of olanzapine has already been conducted in patients with ALS (NCT00876772). However, results have not been published yet.

#### 4 Targeting the 5-HT4R

As microglia and reactive astrocytes are major contributors to ALS pathology, strategies that specifically target these cells have been shown to slow down disease progression.^790^^,^^791^ Besides the 5-HT2R, the 5-HT4R is of interest in modulating neuroinflammation in ALS. Activation of microglial 5-HT4R has been shown to induce the release of exosomes, which may promote inflammatory processes.^782^ In addition, the 5-HT4R is involved in the regulation of astrocyte morphology and function. Our recent study revealed that activation of astrocytic 5-HT4R directly modulates glutamatergic neurotransmission in astrocyte-neuron cocultures.^783^ These data suggest that targeting the 5-HT4R might be beneficial in ALS pathology, but warrant further investigation in disease-related models.

#### 5 Targeting the 5-HT7R

Although the 5-HT7R has not been evaluated as a target for ALS treatment, several studies have demonstrated the importance of 5-HT7R-mediated signaling in various disease-relevant processes, highlighting its therapeutic potential. For example, activation of 5-HT7R by the specific agonists LP44 and LP12 protected neuronal cells from glutamate-induced neurotoxicity via antioxidative and antiapoptotic pathways.^775^^,^^776^ In line with this, the 5-HT7R agonist 8-OH-DPAT increased the surface expression of the AMPAR GluR2 subunit in hippocampal neurons, which has also been suggested to counteract glutamate excitotoxicity.^774^ Application of 5-HT7R inverse agonists, such as SB-269970 and lurasidone, to astrocytes, reduced astroglial glutamate release by downregulating 5-HT7R levels at the plasma membrane,^792^^,^^793^ suggesting that different mechanisms are involved in 5-HT7R-mediated neuroprotection. Targeting the 5-HT7R could be beneficial not only to counteract glutamate excitotoxicity but also to ameliorate mitochondrial dysfunction in ALS, as evidenced by a study showing that systemic treatment with the brain-penetrating 5-HT7R agonist LP-211 rescued defects in mitochondrial function and energy metabolism in a mouse model of Rett syndrome.^794^ Furthermore, multiple studies, including our own, highlight the importance of 5-HT7R activation in neuronal morphology and synaptogenesis, with a particular regulating role in phrenic nerve plasticity.^67^^,^^779^^,^^795^, ^796^, ^797^, ^798^ The 5-HT7R might therefore also be an attractive target to improve breathing dysfunctions in patients with ALS.

Taken together, numerous preclinical studies indicate that targeting the serotonergic system may be beneficial in the symptomatic and disease-modifying treatment of ALS. However, despite promising results in experiments with transgenic ALS models, none of the interventions tested to restore serotonergic deficiencies in patients with ALS have demonstrated therapeutic effects on disease progression in clinical trials.

## VI Conclusion

Currently, available therapies for neurodegenerative diseases provide only symptomatic relief, emphasizing an urgent need for the development of novel therapeutic strategies. This review demonstrated that modulation of the serotonergic system represents a promising strategy that goes beyond the treatment of comorbid symptoms, such as depression and psychosis, to target disease-relevant pathomechanisms and slow down disease progression in individual proteinopathies (Fig. 3, Fig. 4, Fig. 5, Fig. 6). In particular, the role of serotonergic neurotransmission in regulating neuronal plasticity as well as microglial and astrocytic functions is of great importance in combating common hallmarks of neurodegenerative diseases, such as neurodegeneration and neuroinflammation.

Although the serotonergic system has emerged as a promising therapeutic target, none of the proposed strategies has yet been successfully translated into clinical practice. This raises the question: Why have serotonergic therapies largely failed in clinical trials despite their preclinical success?

One plausible reason is the inherent complexity of neurodegenerative diseases in humans compared to preclinical models. Most preclinical studies rely on transgenic animal models with single genetic mutations that reflect only a fraction of human cases. In contrast, the majority of patients develop the disease sporadically due to a combination of genetic risk factors, environmental influences, and age-related vulnerabilities. As a result, therapies that show efficacy in genetically engineered models may not translate to human pathology. Moreover, some animal models fail to accurately replicate the serotonergic imbalances observed in patients, further complicating the predictive value of preclinical research.

Another critical factor is disease timing. Serotonergic dysfunction is known to occur early in the course of the disease, often before overt clinical symptoms. However, most clinical trials enroll patients at advanced stages, when serotonergic deficits are already manifested. Consequently, the therapeutic benefits may be less pronounced or even undetectable. Future trials should therefore prioritize early intervention strategies and incorporate biomarker-based patient selection.

In addition, the heterogeneity of serotonin receptors and transporters involved in different neurodegenerative diseases suggests that a one-size-fits-all approach is unlikely to be successful. Indeed, patient subgroups with distinct serotonergic deficits may respond differently to treatment, necessitating the development of medical approaches that tailor interventions to individual pathophysiological profiles. In this respect, advances in neuroimaging and fluid biomarkers may allow for better patient stratification, thereby improving clinical trial outcomes.

Finally, modulation of defined serotonin receptors may not be sufficient to halt disease progression. Instead, a multimodal approach that combines targeted modulation of the serotonergic system with other disease-modifying strategies may be required. For example, in AD, antibody therapies targeting amyloid and tau pathology could be complemented by 5-HTR ligands to enhance cognitive function and neuroprotection. Similarly, in PD, dopaminergic therapies could be optimized by incorporating serotonergic modulators to mitigate nonmotor symptoms and L-DOPA-induced dyskinesia.

Therefore, future research should prioritize the following areas: (1) refining animal models to more accurately reflect human pathology, (2) optimizing treatment timing by targeting presymptomatic or early-stage patients, (3) improving patient selection through biomarker-driven stratification, and (4) developing combination therapies that integrate serotonergic modulation with other disease-modifying interventions. Addressing these challenges will enhance the translational success of therapies based on serotonergic modulation and unlock their full potential as disease-modifying treatments.

Another interesting aspect for future studies is to validate the role of the serotonergic system in copathologies. Indeed, recent findings have shown that the coexistence of tau, *α*-syn, and TDP-43 aggregates has clinical implications and that the comorbid pathology may be part of the disease progression.^799^^,^^800^ For example, TDP-43 inclusions have been found in AD and patients with PD,^471^^,^^474^^,^^801^ while patients with PD with distinct cognitive symptoms also exhibit amyloid and tau aggregates.^473^^,^^475^ Moreover, abnormal deposition of *α*-syn is frequently seen in a variety of other neurodegenerative proteinopathies, including AD.^150^^,^^151^ Given that an imbalance in the serotonergic system exists in all of the aforementioned disorders, it is intriguing to investigate whether the serotonergic dysfunction may be the driving force behind the comorbidity.

## Conflict of interest

Josephine Labus and Evgeni Ponimaskin are coinventors in the international patent WO/2020/065090 describing the targeting of 5-HT7 receptors for the treatment of tauopathies.
